# Attitudes Toward Seeking Mental Health Services and Mobile Technology to Support the Management of Depression Among Black American Women: Cross-Sectional Survey Study

**DOI:** 10.2196/45766

**Published:** 2023-07-19

**Authors:** Terika McCall, Meagan Foster, Todd A Schwartz

**Affiliations:** 1 Division of Health Informatics Department of Biostatistics Yale School of Public Health New Haven, CT United States; 2 Section of Biomedical Informatics and Data Science Yale School of Medicine New Haven, CT United States; 3 Center for Interdisciplinary Research on AIDS (CIRA) Yale School of Public Health New Haven, CT United States; 4 Department of Biostatistics Gillings School of Global Public Health University of North Carolina at Chapel Hill Chapel Hill, NC United States

**Keywords:** African American, women, depression, telemedicine, mobile health, mHealth, mobile apps, digital health, mental health, gender minority, mobile technology, mobile phone

## Abstract

**Background:**

Depression is a common mental health condition among Black American women. Many factors may contribute to the development of depressive symptoms, such as gender and racial discrimination, financial strain, chronic health conditions, and caregiving responsibilities. Barriers such as the stigmatization of mental illness, less access to treatment, the lack of or inadequate health insurance, mistrust of providers, and limited health literacy prevent marginalized populations from seeking care. Previous literature has shown that mobile health interventions are effective and can increase access to mental health services and resources.

**Objective:**

We aimed to understand the attitudes and perceptions of Black women toward using mental health services and determine the acceptability and concerns of using mobile technology (ie, voice call, video call, SMS text messaging, and mobile app) to support the management of depression.

**Methods:**

We launched a self-administered web-based questionnaire in October 2019 and closed it in January 2020. Women (aged ≥18 years) who identify as Black or African American or multiracial (defined as Black or African American and another race) were eligible to participate. The survey consisted of approximately 70 questions and included topics such as attitudes toward seeking professional psychological help, the acceptability of using a mobile phone to receive mental health care, and screening for depression.

**Results:**

The findings (n=395) showed that younger Black women were more likely to have greater severity of depression than their older counterparts. The results also revealed that Black women have favorable views toward seeking mental health services. Respondents were the most comfortable with the use of voice calls or video calls to communicate with a professional to receive support for managing depression in comparison with SMS text messaging or mobile apps. The results revealed that higher help-seeking propensity increased the odds of indicating agreement with the use of voice calls and video calls to communicate with a professional to receive support for managing depression by 27% and 38%, respectively. However, no statistically significant odds ratios (all *P*>.05) were found between help-seeking propensity and respondents’ agreement to use mobile apps or SMS text messaging. Moderate to severe depression severity increased the odds of using mobile apps to communicate with a professional to receive support for managing depression by 43%; however, no statistically significant odds ratios existed for the other modalities. Privacy and confidentiality, communication issues (eg, misinterpreting text), and the impersonal feeling of communicating by mobile phone (eg, SMS text messaging) were the primary concerns.

**Conclusions:**

Black American women, in general, have favorable views toward seeking mental health services and are comfortable with the use of mobile technology to receive support for managing depression. Future work should address the issues of access and consider the preferences and cultural appropriateness of the resources provided.

## Introduction

### Background

Mood disorders, such as major depressive disorder, are among the most common mental health conditions among Black American women [1]. An estimated 27% of non-Hispanic Black women experience depression in their lifetime [2]. Predictive factors such as gender and racial discrimination, lower socioeconomic status, cardiometabolic conditions (such as hypertension, diabetes, and obesity), and other complex life demands (such as stress from multigenerational caregiving) exist at high rates among Black women and increase the risk of psychological distress and depressive symptoms [3-8]. The COVID-19 pandemic may have further increased the prevalence estimates owing to the effects of structural gendered racism on Black women’s mental health and factors caused or worsened by pandemic-related stressors, which have disproportionately affected the Black community [9]. Empowering archetypes deeply held by Black American women such as the Superwoman Schema [10] and Strong Black Woman Schema [11] can both positively and adversely influence attitudes toward seeking professional psychological services and result in harmful coping mechanisms, such as suppressed emotional responses and self-silencing [10,12-14]. These findings further emphasize the need for culturally tailored diagnostic and treatment tools for Black American women dealing with depression.

Barriers such as the stigmatization of mental illness, less access to treatment, the lack of or inadequate health insurance, mistrust of providers, and limited health literacy prevent marginalized populations from seeking care [15-17]. Disparities in mental health service use are concerning because poor management of depressive symptoms contributes to a lower quality of life, loss of productivity, and poor medication adherence, which may lead to worse health outcomes [18-20]. Past interventions using modalities such as voice calls [21,22], video calls [23,24], SMS text messaging [25,26], and mobile apps [27-30] were effective in reducing depressive symptoms. Furthermore, the use of mobile technology may help mitigate barriers to receiving support by providing information on affordable options for mental health care, facilitating connections with preferred therapists (eg, shared identities, such as race and sexual orientation), eliminating travel time through the use of remote services, and may lessen stigma by providing a more discreet way to receive care [31-33].

Black women have high rates of smartphone ownership (80%) and, in general, are comfortable with participating in mobile health (mHealth) research and interventions [34,35]. Moreover, they are more willing to participate in mHealth research “if they were interested in the topic, if they had an opportunity to become more educated about the topic, and to contribute to the greater good” [35]. However, most of the published studies examining the use of mHealth interventions to support the management of depression were conducted with predominantly White participants. Therefore, the results may not be generalizable to all racial groups. Further exploration of the use of mobile technology to provide mental health resources and services to Black American women is necessary.

Building upon prior work [34,36], the purpose of this study was to gauge the attitudes and perceptions of Black American women toward using mental health services and determine the acceptability of using mobile technology (eg, mobile app, video call, voice call, or SMS text messaging) to support the management of depression. The study was guided by two theoretical frameworks: (1) the Theory of Planned Behavior (TPB) model [37] and (2) the Technology Acceptance Model (TAM) [38]. The TPB model [37] expands on the Theory of Reasoned Action by Fishbein and Ajzen [39] by including the concept of perceived behavioral control. Ajzen [37] proposed that attitude toward behavior, subjective norm, and perceived behavioral control collectively forms one’s behavioral intentions and behaviors. In the TPB model, attitude refers to an individual’s positive or negative assessment of the behavior (eg, going to therapy is good), subjective norm refers to perceived pressure from others to perform the behavior (eg, my friends would want me to go to therapy), and perceived behavioral control refers to one’s beliefs about the ease or difficulty of performing the behavior (eg, it would be easy for me to go to therapy) [37,40]. Perceived behavioral control can also directly influence behavior. The TAM is a theory that suggests perceived usefulness and perceived ease of use of a new technology impact attitude toward using the technology, which in turn influences how users come to accept (ie, behavioral intention to use) and use (ie, actual system use) new technology [38]. The 2 key components of the model, perceived usefulness and perceived ease of use, are affected by external variables such as system design characteristics. “A key purpose of TAM, therefore, is to provide a basis for tracing the impact of external factors on internal beliefs, attitudes, and intentions” [41].

### Objectives

The following research questions were examined: (1) What are the attitudes and perceptions of Black women toward using mental health services? (2) Is the use of mobile technology acceptable to Black women to receive support to manage depression? (3) Are there modalities that are more acceptable than others (eg, mobile app, video call, voice call, or SMS text messaging) to use to deliver mental health services and resources to Black women to support the management of depression? and (4) What are the primary concerns about using mobile technology to receive mental health treatment or counseling?

## Methods

### Study Design and Recruitment

We launched the self-administered web-based questionnaire in October 2019 and closed it in January 2020. For the purpose of this study, the classification of “African American,” “Black,” or “Black American” refers to individuals who noted their race as such, are a part of the African diaspora, and reside in the United States. Women (aged ≥18 years) who identify as Black or African American or multiracial (defined as Black or African American and another race) and residing in the United States were eligible to participate. The respondents were recruited through convenience sampling. Recruitment methods included receiving an invitation to take the survey via an anonymous link distributed through listserves whose membership is primarily Black American women (eg, National Council of Negro Women, Inc), university student group listserves, and church membership listserves. Respondents were also recruited via posts on social media (eg, Facebook and Twitter). Following a snowball sampling technique, respondents were allowed to share the link to the survey with their networks (eg, family and friends).

Sample proportions from the preliminary study exploring the acceptability of telemedicine to support the management of anxiety and depression among Black American women were considered for use in nQuery (Statsols) statistical software to determine the necessary sample size to obtain a specified level of precision via a half-width of 0.05 for a 2-sided 95% CI [34,36]. On the basis of a conservative scenario with an expected proportion of 0.50, the sample size required to obtain this level of precision was determined to be 385.

### Measures

#### Overview

The Institutional Review Board of the University of North Carolina at Chapel Hill approved the use of the *Attitudes Toward Seeking Mental Health Services and Use of Mobile Technology Survey*. The survey consisted of approximately 70 questions and was administered using the Qualtrics (SAP) software. Survey domains included (1) sociodemographic characteristics, (2) attitudes toward seeking professional psychological help (questions from an adapted version of the validated Inventory of Attitudes Toward Seeking Mental Health Services [IASMHS]), (3) mobile phone use, (4) the acceptability of using a mobile phone to receive mental health care, (5) screening for the presence and severity of depression using the Patient Health Questionnaire 9-item scale (PHQ-9) [42], (6) past mental health services use using questions from the *2019 National Survey on Drug Use and Health* [43], and (7) a history of mental illness. Respondents were informed that they can choose not to answer any question they do not wish to answer. They could also choose to stop taking the survey at any time. See Multimedia Appendix 1 for the survey instrument.

Questions on sociodemographic characteristics, such as the respondent’s race, ethnicity, age, gender, highest level of education attained, and annual household income, were asked at the beginning of the survey. The race, age, and gender questions were used as screener questions to determine eligibility to continue the survey. If the respondent did not self-identify as Black or African American or multiracial (defined as Black or African American and another race) and was ≤18 years, they were routed directly to the end of the survey. In addition, if the respondent self-identified as male, they were routed directly to the end of the survey. To prevent false responses from bots, the aforementioned screener questions were used in the skip logic for the web-based questionnaire (routed to the end of the survey), and the options to prevent multiple submissions and prevent indexing were active. During data collection, the first author reviewed the data at least twice per week for suspicious responses.

#### Depression Screening

If the respondents passed the screening questions, they were permitted to complete the rest of the survey. The next set of questions included the PHQ-9 [42] screen for the presence and severity of depression. The PHQ-9 is a 9-item measure evaluating the presence and severity of depression. The scale ranges from a score of 0 to 27. A score of ≥10 on the PHQ-9 represents a reasonable cutoff point for identifying depressive disorders [42]. Scores of 0 to 4 indicate a *minimal* level of depression, 5 to 9 indicate a *mild* level of depression, 10 to 14 indicate a *moderate* level of depression, 15 to 19 indicate a *moderately* severe level of depression, and 20 to 27 indicate a *severe* level of depression [42].

#### Attitudes Toward Seeking Professional Psychological Help

Respondents’ attitudes toward seeking professional psychological help were measured using questions from an adapted version of the validated IASMHS [44]. The IASMHS consists of 24 questions that contribute to a total IASMHS score and the following factors: psychological openness (ie, the extent to which individuals are open to acknowledging psychological problems and to the possibility of seeking professional help for them), help-seeking propensity (ie, the extent to which individuals believe they are willing and able to seek professional psychological help), and indifference to stigma (ie, the extent to which individuals are concerned about what various important others might think should they find out that the individual were seeking professional help for psychological problems). The response options to the survey items were on a 5-point Likert-type scale ranging from 0 (disagree) to 4 (agree). Before the data analysis, all negatively worded items were reverse coded.

In the survey, the term *professional* refers to individuals who are trained mental health specialists (eg, psychologists, psychiatrists, social workers, and family physicians). To collect data specifically on attitudes toward seeking professional help for managing depression, 6 questions in the inventory were revised. In these 6 questions, the words *psychological problems* or *mental disorder* were substituted with *depression*. For example, item 16 in the IASMHS reads, “I would be uncomfortable seeking professional help for psychological problems because people in my social or business circles might find out about it.” The corresponding revised survey question states, “I would be uncomfortable seeking professional help for *depression* because people in my social or business circles might find out about it.” This increased the total number of questions in the inventory to 30 and permitted the calculation of a total IASMHS score related to depression and subscores for psychological openness, help-seeking propensity, and indifference to stigma for depression. Scores on the IASMHS ranged from 0 to 96, with subscale scores ranging from 0 to 32. Higher scores indicate more positive attitudes toward seeking professional psychological help.

#### Mental Health Service Use

Data on past mental health service use and barriers to receiving treatment despite perceived need were collected through the use of questions from the *2019 National Survey on Drug Use and Health* [43]. Respondents were given instructions that the questions were about treatment and counseling for problems with emotions, nerves, or mental health. They were also asked not to include treatment for alcohol or drug use.

#### Mobile Phone Use

Mobile phone use was ascertained with the following items: (1) current mobile phone ownership (yes or no), (2) the frequency of sending SMS text messages (never, <1 time/wk, 1-6 times/wk, 1-3 times/d, and ≥4 times/d), (3) the frequency of accessing apps on a phone (never, <1 time/wk, 1-6 times/wk, 1-3 times/d, and ≥4 times/d), (4) the ability to complete video calls on a mobile phone (yes or no), and (5) the frequency of using a mobile phone to complete video calls (never, <1 time/wk, 1-6 times/wk, 1-3 times/d, and ≥4 times/d).

#### The Acceptability of Mobile Phone Use for Mental Health Care

The acceptability of using a mobile phone to receive mental health care to manage depression was measured by comfortability communicating with a professional through SMS text messaging, voice call, mobile app, or video call to receive help for managing depression. Moreover, the following statement was presented to further gauge the acceptability of using the modalities: “Having the option to use [text messaging/voice call/mobile app/video call] to communicate with a professional if I am dealing with depression would be helpful for me.”

The response options to the survey items were on a 5-point Likert-type scale ranging from 1 (disagree) to 5 (agree). Before completing the survey, respondents were asked, “Do you have any concerns about using a [voice call/mobile app/video call] to receive mental health treatment or counseling?” and “Do you have any concerns about using text messaging to communicate with a professional about your mental health?” If they answered “Yes” to these questions, they were presented with open-ended questions asking them to note their concerns in the textboxes provided.

#### Mental Health History

The final section of the survey asked questions about past diagnoses of depression. Respondents were also asked if they currently have health insurance.

### Statistical Analysis

#### Quantitative Data Analysis

Descriptive statistics were calculated as means, SDs, and ranges for continuous variables and as frequencies and percentages for categorical variables for sample characteristics and responses to questions about the use of the modalities (eg, video call) to receive mental health services. The age variable was grouped (18-24, 25-34, 35-49, 50-64, and ≥65 years) according to results from a prior study on the mental health of Black women in the United States that used data from a national survey [1]. Demographic variables also included race (Black or African American or multiracial—defined as Black or African American and another race), ethnicity (Hispanic or non-Hispanic), education (high school diploma or General Educational Development tests; some college, <4-year degree; or bachelor’s degree or higher), income (≤US $10,000, US $10,000-US $24,999, US $25,000-US $49,999, US $50,000-US $100,000, or ≥US $100,000), health insurance (yes or no), and the region in the United States in which the respondent currently lives (Midwest, Northeast, West, or South). For additional context, age was dichotomized into 2 groups (<50 years and ≥50 years) and education was categorized into 2 levels (less than a bachelor’s degree and a bachelor’s degree or higher) to be consistent with the preliminary study [34,36].

Response options were dichotomized as agreed (*agree or somewhat agree*) versus those who did not indicate agreement (*disagree, somewhat disagree, or undecided*). The Fisher exact test was used to (1) determine whether a statistically significant association exists between responses to questions about comfortability with using each modality (SMS text messaging, voice call, mobile app, or video call) to communicate with a professional to receive help for managing depression (agreed vs those who did not indicate agreement) with age group and education level, respectively, and (2) test for an association between agreement (agreed vs those who did not indicate agreement) with response to questions about comfortability and perceived helpfulness of having the option to communicate with a professional, using mobile technology, to receive help for managing depression. Independent groups 2-tailed *t* tests were performed separately to assess group differences in mean scores for PHQ-9, psychological openness, help-seeking propensity, indifference to depression stigma, and IASMHS scores for depression between the groups of respondents who agreed with the use of various modalities to communicate with a professional to receive help to manage depression versus those who did not indicate agreement. Missing responses were excluded from the analysis.

Multivariable logistic regression modeling was performed to estimate odds ratios (ORs) and their corresponding 95% CIs to assess the strength of the association between the responses to questions about comfortability with using each modality to communicate with a professional to receive help for managing depression (agreed vs those who did not indicate agreement), with education level, household income, health insurance, PHQ-9, psychological openness, help-seeking propensity, indifference to depression stigma, past mental health service use, unmet mental health need, and region as the explanatory variables. These models examined the associations between responses to questions about comfortability with using each modality to communicate with a professional to receive help for managing depression (agreed vs those who did not indicate agreement), with PHQ-9, psychological openness, help-seeking propensity, and indifference to depression stigma as explanatory variables, managed both as a continuous effect (assuming linearity) and dichotomized into high versus low (with no such assumption). Age and history of depression were used as control variables. Statistical significance was determined at the 2-sided .05 significance level for all tests; no adjustments were made for multiple comparisons.

To assess whether there was effect modification for Black women with low to mild depression severity (who may not have pressing concerns about their mental health) versus those with higher levels (who may screen positive) on the multivariably adjusted ORs, we separately tested the interactions between the depression severity cutoff scores of 7 and 10 with each psychological barrier, such as depression stigma, psychological openness, and help-seeking propensity, for respondents’ comfort using each modality. Statistical analyses were conducted using SPSS (IBM Corp) and SAS (SAS Institute) statistical software.

#### Qualitative Data Analysis

In addition, thematic analysis—a method for identifying, analyzing, and reporting patterns (themes) within data—was conducted on responses to the questions, “What are your concerns about using a [voice call/mobile app/video call] to receive mental health treatment or counseling?” and “What are your concerns about using text messaging to communicate with a professional about your mental health?” The responses were imported into NVivo (QSR International) software for analysis. The data were categorized by the first author (TM) by reading through each response and coding the emerging themes. Responses were assigned as many themes as were pertinent. The results of the thematic analysis were independently reviewed (a professor with a PhD in Health Informatics) to ensure reliability and consistency in coding.

### Ethical Considerations, Informed Consent, and Data Privacy

The Institutional Review Board of the University of North Carolina at Chapel Hill provided the study a notification of exemption from further review. Detailed information about the study is provided on the first screen of the survey. The respondents were informed that completing the survey signified consent; however, they can choose to discontinue the survey at any time. An anonymous link was provided to access the survey to protect the privacy and confidentiality of respondents. No personally identifiable information was collected during the survey. The survey responses were collected and stored separately from the respondents’ identifiers in Health Insurance Portability and Accountability Act–compliant cloud storage. Respondents who completed the survey were eligible to provide their contact information for entry into a drawing to receive 1 of 5 e-gift cards worth US $100 each. Participation in the drawing was optional. A separate questionnaire was used to collect contact information for the drawing and for the notification of an opportunity to participate in a focus group (respondents could opt in to both). The questionnaire was linked to the end of the main survey.

## Results

### Participants

#### Overview

The results of this study focus on Black American women’s attitudes toward the use of mobile technology to support the management of depression. The sociodemographic characteristics of the survey respondents are summarized in Table 1. Of the 491 respondents who started the survey, 395 (80.4% completion rate) respondents completed it. Respondents ranged in age from 18 to 107 (mean age 44.8, SD 18.4) years, and all identify as either Black or African American or multiracial (ie, Black or African American and another race) and female. Most respondents (380/395, 96.2%) identify as non-Hispanic. Furthermore, 312 (79%) out of 395 respondents had a bachelor’s degree or higher. The annual household income was reported to be ≤US $50,000 for 40.3% (159/395) of respondents, US $50,000-US $100,000 for 34.9% (138/395) of respondents, and ≥US $100,000 for 23.8% (94/395) of respondents. The majority (371/395, 93.9%) indicated that they had health insurance.

**Table 1:** Sociodemographic characteristics of the survey respondents (n=395).

Characteristics			Values
Age (years), mean (SD)			44.8 (18.4)
**Age range (years), n (%)**			
	18-24	59 (14.9)	
	25-34	98 (24.8)	
	35-44	46 (11.6)	
	45-54	58 (14.7)	
	55-64	55 (13.9)	
	≥65	79 (20)	
**Age group (years), n (%)**			
	<50	232 (58.7)	
	≥50	163 (41.3)	
**Race, n (%)**			
	Black or African American	377 (95.4)	
	Multiracial^a^	18 (4.6)	
**Ethnicity, n (%)**			
	Hispanic	15 (3.8)	
	Non-Hispanic	380 (96.2)	
**Education, n (%)**			
	High school diploma or GED^b^	13 (3.3)	
	Some college, <4-year degree	70 (17.7)	
	Bachelor’s degree or higher	312 (79)	
**Education group, n (%)**			
	Less than a bachelor’s degree	83 (21)	
	Bachelor’s degree or higher	312 (79)	
**Income (US $)^c^, n (%)**			
	<10,000	30 (7.6)	
	10,000-24,999	37 (9.4)	
	25,000-49,999	92 (23.3)	
	50,000-100,000	138 (34.9)	
	>100,000	94 (23.8)	
**Health insurance^d^, n (%)**			
	Yes	371 (93.9)	
	No	23 (5.8)	
**Region, n (%)**			
	Midwest	60 (15.19)	
	Northeast	69 (17.47)	
	West	34 (8.61)	
	South	229 (57.97)	

^a^Multiracial was defined as identifying as Black or African American and another race.

^b^GED: General Educational Development tests.

^c^The total sample size <395 and percentages may not sum up to 100% because of item missingness (n=4) and rounding.

^d^The total sample size <395 and percentages may not sum up to 100% because of item missingness (n=1) and rounding.

#### Mobile Phone Use

The survey respondents’ mobile phone use is summarized in Table 2. The vast majority (391/395, 99%) indicated that they have a mobile phone. Respondents who indicated that they did not own a mobile phone were not presented with questions on their mobile phone use. Most participants (388/395, 98.2%) reported the use of SMS text messaging, and 72.7% (287/395) indicated texting ≥4 times per day. Most respondents (381/395, 96.4%) indicated that they used mobile apps, and 73.4% (290/395) indicated that they used a mobile app ≥4 times per day. Most (358/395, 90.6%) indicated that their phones had video call capability. Respondents who indicated that their phones did not have video call capability were not presented with the frequency of use question. Most respondents (318/395, 80.5%) reported on the use of video call, and 38.4% (152/395) indicated using video call at least once per week.

**Table 2:** Mobile phone use (n=395).

Characteristic		Values
**Mobile phone ownership, n (%)**		
	Yes	391 (99)
	No	4 (1)
**Frequency of sending SMS text messages^a^, n (%)**		
	Never	3 (0.8)
	<1 time/wk	9 (2.3)
	1-6 times/wk	36 (9.1)
	1-3 times/d	56 (14.2)
	≥4 times/d	287 (72.7)
**Frequency of accessing mobile apps^a^, n (%)**		
	Never	10 (2.5)
	<1 time/wk	16 (4.1)
	1-6 times/wk	29 (7.3)
	1-3 times/d	46 (11.6)
	≥4 times/d	290 (73.4)
**Mobile phone video call capability^a^, n (%)**		
	Yes	358 (90.6)
	No	33 (8.4)
**Frequency of video call use^a,b^, n (%)**		
	Never	40 (10.1)
	<1 time/wk	166 (42)
	1-6 times/wk	98 (24.8)
	1-3 times/d	27 (6.8)
	≥4 times/d	27 (6.8)

^a^Respondents who indicated that they did not own a mobile phone (n=4) were not presented with questions on mobile phone use.

^b^Respondents who indicated that their phone did not have video call capability (n=33) were not presented with the frequency of use question.

#### Depression Severity

**Figure 1:**
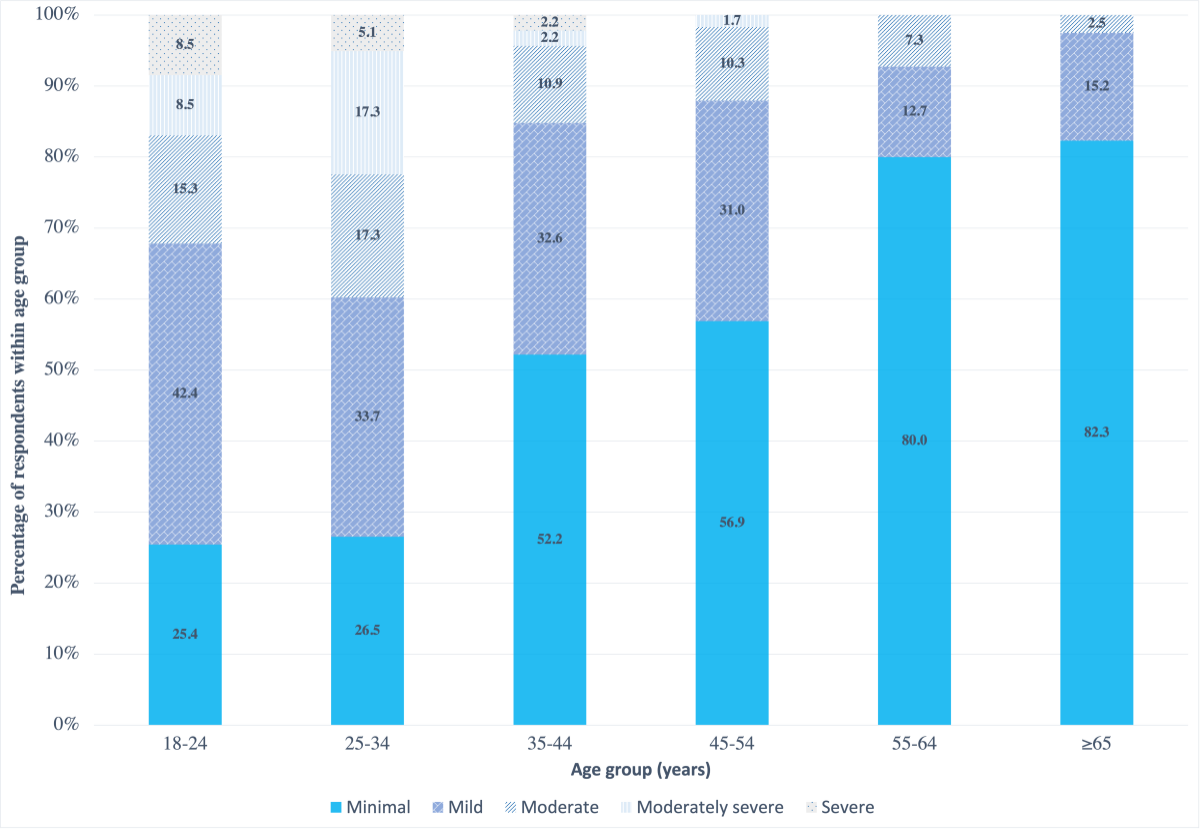
Sample percentages for depression severity by age group. Total percentages may not sum up to 100% owing to item missingness.

Almost one-third (117/395, 29.6%) of the respondents reported being diagnosed with depression in the past. Questions from the PHQ-9 were used to screen for current presence and severity of depression [42]. The mean score for depression severity (PHQ-9) was 5.72 (SD 5.51). The sample percentages for depression severity by age group are shown in Figure 1. The 25 to 34 years age group had the highest percentage of individuals with depression, reporting 17% (17/98) of respondents with a moderate level of depression, 17% (17/98) of respondents with a moderately severe level of depression, and 5% (5/98) of respondents with a severe level of depression. Respondents in the 18 to 24 years age group had the next highest percentage of individuals with depression, reporting 15% (9/59) of respondents with a moderate level of depression, 9% (5/59) of respondents with a moderately severe level of depression, and 9% (5/59) of respondents with a severe level of depression. The 35 to 44 years age group reported 11% (5/46) of respondents with a moderate level of depression, 2% (1/46) of respondents with a moderately severe level of depression, and 2% (1/46) of respondents with a severe level of depression. The 45 to 54 years age group reported 10% (6/58) of respondents with a moderate level of depression, 2% (1/58) of respondents with a moderately severe level of depression, and no respondents with a severe level of depression. The 55 to 64 years age group reported 7% (4/55) of respondents with a moderate level of depression and no respondents with a moderately severe or severe level of depression. Finally, the ≥65 years age group reported 3% (2/79) of respondents with a moderate level of depression and no respondents with a moderately severe or severe level of depression.

### Attitudes Toward Seeking Mental Health Services

The respondents held favorable views toward seeking mental health services. Higher scores indicated more positive attitudes toward seeking professional psychological help (ranging from 0 to 32). The mean score for psychological openness was 23.53 (SD 5.52), and the mean score for help-seeking propensity was 25.91 (SD 5.53). The mean score for indifference to depression stigma was 24.50 (SD 6.56).

### The Acceptability of Mobile Technology to Support the Management of Depression

**Figure 2:**
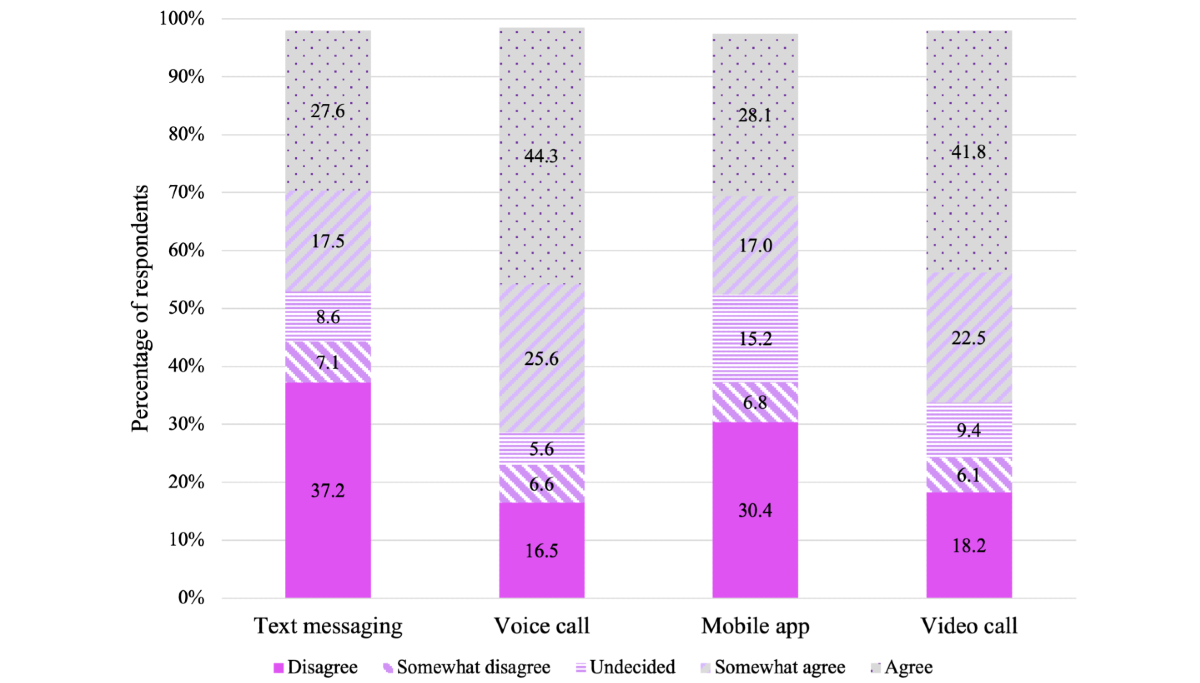
Sample percentages, by modality, for response to the statement, “I would feel comfortable communicating with a professional through [modality] to receive help for managing depression.”.

The results of the survey revealed that respondents were most comfortable with the use of voice call followed by video call, mobile app, and SMS text messaging to communicate with a professional to receive help for managing depression (Figure 2). Most respondents (276/395, 69.9%) indicated agreement (175/395, 44.3% agree and 101/395, 25.6% somewhat agree), 5.6% (22/395) were undecided, and 23.1% (91/395) showed disagreement (65/395, 16.5% disagree and 26/395, 6.6% somewhat disagree) with the use of a voice call to receive help in managing depression. Furthermore, 64.3% (254/395) of respondents indicated agreement (165/395, 41.8% agree and 89/395, 22.5% somewhat agree), 9.4% (37/395) were undecided, and 24.3% (96/395) showed disagreement (72/395, 18.2% disagree and 24/395, 6.1% somewhat disagree) with the use of video call to receive help for managing depression. Nearly half (178/395, 45.1%) of the respondents indicated agreement (111/395, 28.1% agree and 67/395, 17% somewhat agree), 15.2% (60/395) were undecided, and 37.2% (147/395) showed disagreement (120/395, 30.4% disagree and 27/395, 6.8% somewhat disagree) with the use of a mobile app to receive help for managing depression. Similarly, 45.1% (178/395) of respondents indicated agreement (109/395, 27.6% agree and 69/395, 17.5% somewhat agree), 8.6% (34/395) were undecided, and 44.3% (175/395) showed disagreement (147/395, 37.2% disagree and 28/395, 7.1% somewhat disagree) with the use of SMS text messaging to receive help for managing depression.

**Figure 3:**
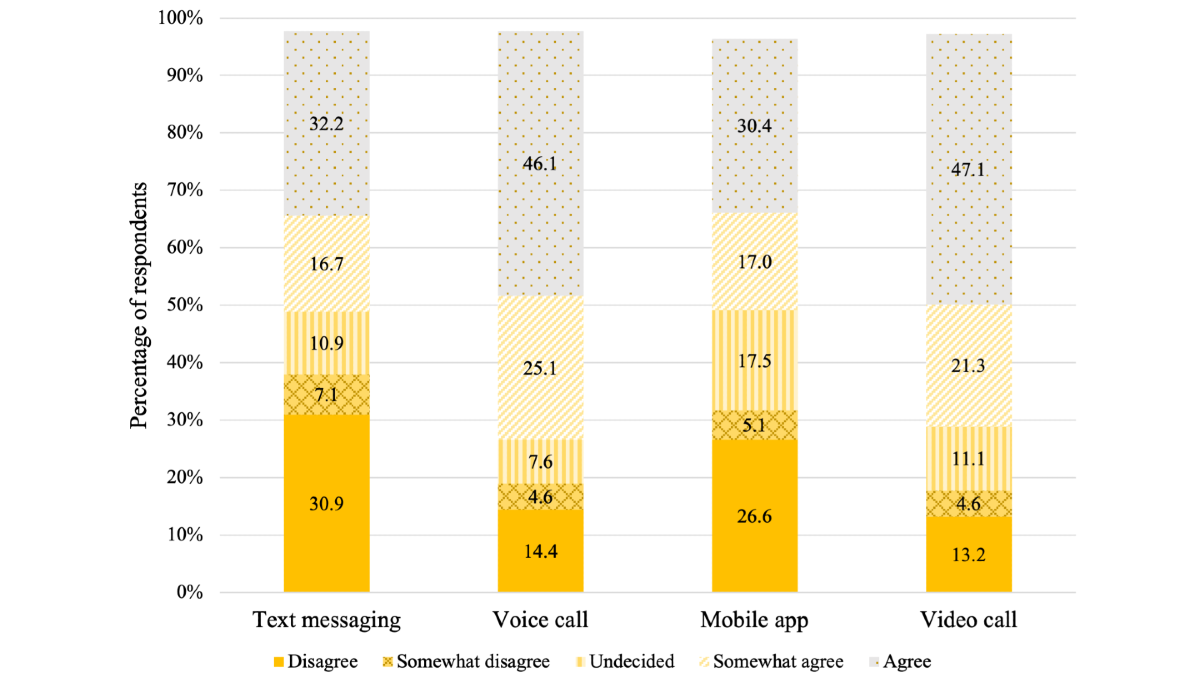
Sample percentages, by modality, for response to the statement, “Having the option to use [modality] to communicate with a professional if I am dealing with depression would be helpful for me.”.

Most respondents (281/395, 71.2%) agreed (182/395, 46.1% agree and 99/395, 25.1% somewhat agree) that having the option to use voice call to communicate with a professional if they are dealing with depression would be helpful. Furthermore, 68.4% (268/395) of respondents agreed (184/395, 47.1% agree and 84/395, 21.3% somewhat agree) that a video call option would be helpful, 48.9% (193/395) agreed (127/395, 32.2% agree and 66/395, 16.7% somewhat agree) that an SMS text messaging option would be helpful, and 47.4% (187/395) agreed (120/395, 30.4% agree and 67/395, 17% somewhat agree) that a mobile app option would be helpful (Figure 3). Statistically significant associations were found between agreement with comfortability (agree or somewhat agree) and perceived helpfulness with having the option to communicate with a professional through SMS text messaging (165/191, 86.4% were comfortable among those that felt it would be helpful vs 11/191, 5.8%, who were comfortable among those that felt it would *not* be helpful; *P*<.001), voice call (258/281, 91.8% were comfortable among those that felt it would be helpful vs 16/105, 15.2%, who were comfortable among those that felt it would *not* be helpful; *P*<.001), mobile app (160/185, 86.5% were comfortable among those that felt it would be helpful vs 15/193, 7.8%, who were comfortable among those that felt it would *not* be helpful; *P*<.001), and video call (237/269, 88.15% were comfortable among those that felt it would be helpful vs 16/113, 14.2%, who were comfortable among those that felt it would *not* be helpful; *P*<.001) to receive help for managing depression.

Statistically significant associations were found between age (≤50 years vs ≥50 years) and agreement (agree or somewhat agree) with the use of SMS text messaging (125/232, 53.9% agreed vs 53/163, 32.5% agreed, respectively; *P*<.001), voice call (174/232, 75% agreed vs 102/163, 62.6% agreed, respectively; *P*=.04), mobile app (131/232, 56.5% agreed vs 47/163, 28.8% agreed, respectively; *P*<.001), and video call (168/232, 72.4% agreed vs 86/163, 52.8% agreed, respectively; *P*=.001) to communicate with a professional to receive help for managing depression (Table 3). Furthermore, statistically significant associations were found between education (less than a bachelor’s degree vs a bachelor’s degree or higher) and agreement (agree or somewhat agree) with the use of SMS text messaging (46/83, 55% agreed vs 132/312, 42.3% agreed, respectively; *P*=.03) and mobile app (45/83, 54% agreed vs 133/312, 42.6%agreed, respectively; *P*=.045) to communicate with a professional to receive help for managing depression. No statistically significant associations were found between education (less than a bachelor’s vs a bachelor’s degree or higher) and agreement (agree or somewhat agree) with the use of voice call (58/83, 70% agreed vs 218/312, 69.9%, respectively; *P*>.99) and video call (52/83, 63% agreed vs 202/312, 64.7% agreed, respectively; *P*=.79) to communicate with a professional to receive help for managing depression (Table 4).

**Table 3:** Agreement with the use of modality to communicate with a professional to receive help for managing depression by age group^a^.

Modality^b^	<50 years (n=232), n (%)			≥50 years (n=163), n (%)			Fisher exact *P* value	
	Agree	Did not agree	Agree		Did not agree			
SMS text messaging	125 (53.9)	107 (46.1)	53 (32.5)		102 (62.6)	*<.001* ^c^		
Voice call	174 (75)	58 (25)	102 (62.6)		55 (33.7)	*.04*		
Mobile app	131 (56.5)	101 (43.5)	47 (28.8)		106 (65)	*<.001*		
Video call	168 (72.4)	64 (27.6)	86 (52.8)		69 (42.3)	*.001*		

^a^“Undecided” responses were combined with “disagree” and “somewhat disagree” responses to form a “did not agree” category for those who did not indicate agreement.

^b^The total sample size <395 and percentages may not sum to 100% owing to item missingness.

^c^Italicized *P* value denotes statistical significance.

**Table 4:** Agreement with the use of modality to communicate with a professional to receive help for managing depression by education^a^.

Modality^b^	Less than a bachelor’s degree (n=83), n (%)		A bachelor’s degree or higher (n=312), n (%)		Fisher exact *P* value
	Agree	Did not agree	Agree	Did not agree	
SMS text messaging	46 (55.4)	35 (42.2)	132 (42.3)	174 (55.8)	*.03* ^c^
Voice call	58 (69.9)	23 (27.7)	218 (69.9)	90 (28.8)	>.99
Mobile app	45 (54.2)	35 (42.2)	133 (42.6)	172 (55.1)	*.04*
Video call	52 (62.7)	29 (34.9)	202 (64.7)	104 (33.3)	.79

^a^“Undecided” responses were combined with “disagree” and “somewhat disagree” responses to form a “did not agree” category for those who did not indicate agreement.

^b^The total sample size <395 and percentages may not sum to 100% owing to item missingness.

^c^Italicized *P* value denotes statistical significance.

### Attitudes Toward Seeking Help by Agreement With Modality

The results of the study showed statistically significant differences between group mean scores for PHQ-9 (mean 6.7, SD 6.1 vs mean 5.0, SD 4.9, respectively; *P*=.003) between the respondents who agreed to the use of SMS text messaging to communicate with a professional to receive help for managing depression and those who did not indicate agreement. No statistically significant differences were found between group mean scores for psychological openness (mean 23.2, SD5.7 vs mean 23.8, SD 5.4, respectively; *P*=.27), help-seeking propensity (mean 25.8, SD 5.2 vs mean 25.9, SD 5.9, respectively; *P*>.99), indifference to depression stigma (mean 23.9, SD 6.9 vs mean 24.9, SD 6.3, respectively; *P*=.15), and IASMHS total score (mean 72.9, SD 14.5 vs mean 74.5, SD 13.9, respectively; *P*=.28) between the respondents who agreed with the use of SMS text messaging to communicate with a professional to receive help for managing depression and those who did not indicate agreement (Table 5). Statistically significant differences were found between group mean scores for help-seeking propensity (mean 26.5, SD 4.9 vs mean 24.3, SD 6.6, respectively; *P*=.001) and IASMHS total score (mean 74.9, SD 13.5 vs mean 70.9, SD 15.5, respectively; *P*=.02) between the respondents who agreed with the use of voice call to communicate with a professional to receive help for managing depression and those who did not indicate agreement. However, no statistically significant differences were found between group mean scores for PHQ-9 (mean 5.8, SD 5.5 vs mean 5.7, SD 5.6, respectively; *P*=.87), psychological openness (mean 23.6, SD 5.6 vs mean 23.3, SD 5.3, respectively; *P*=.56), and indifference to depression stigma (mean 24.8, SD 6.3 vs mean 23.6, SD 7.2, respectively; *P*=.13) between the respondents who agreed with the use of voice call to communicate with a professional to receive help for managing depression and those who did not indicate agreement (Table 6).

Moreover, statistically significant differences were found between group mean scores for PHQ-9 (mean 7.0, SD 6.3 vs mean 4.8, SD 4.6, respectively; *P*<.001) between the respondents who agreed with the use of mobile app to communicate with a professional to receive help for managing depression and those who did not indicate agreement. No statistically significant differences were found between group mean scores for psychological openness (mean 23.1, SD 5.9 vs mean 23.8, SD 5.2, respectively; *P*=.22), help-seeking propensity (mean 25.9, SD 5.6 vs mean 25.8, SD 5.6, respectively; *P*=.96), indifference to depression stigma (mean 24.4, SD 6.7 vs mean 24.4, SD 6.5, respectively; *P*=.93), and IASMHS total score (mean 73.3, SD 14.7 vs mean 73.9, SD 13.8, respectively; *P*=.67) between the respondents who agreed with the use of mobile app to communicate with a professional to receive help for managing depression and those who did not indicate agreement (Table 7). Statistically significant differences were found between group mean scores for psychological openness (mean 24.1, SD 5.5 vs mean 22.5, SD 5.6, respectively; *P*=.008), indifference to depression stigma (mean 25.0, SD 6.2 vs mean 23.4, SD 7.2, respectively; *P*=.03), and IASMHS total score (mean 75.2, SD 13.3 vs mean 70.8, SD 15.4, respectively; *P*=.005) between the respondents who agreed with the use of video call to communicate with a professional to receive help for managing depression and those who did not indicate agreement. However, no statistically significant differences were found between group mean scores for PHQ-9 (mean 6.0, SD 5.6 vs mean 5.3, SD 5.4, respectively; *P*=.24) and help-seeking propensity (mean 26.3, SD 5.2 vs mean 25.1, SD 6.1, respectively; *P*=.053) between the respondents who agreed with the use of video call to communicate with a professional to receive help for managing depression and those who did not indicate agreement (Table 8).

**Table 5:** The Patient Health Questionnaire 9-item scale (PHQ-9) and Inventory of Attitudes Toward Seeking Mental Health Services (IASMHS) factor scores by agreement with using SMS text messaging to communicate with a professional to receive help for managing depression^a^.

Measure^b^	Agree			Did not agree			*t* test (*df*)		*P* value
	Values, n (%)	Values, mean (SD)	Values, n (%)		Values, mean (SD)				
PHQ-9 (n=384)	176 (44.6)	6.7 (6.1)	208 (52.7)		5.0 (4.9)	2.98 (335.09)		*.003* ^c^	
Psychological openness (n=386)	178 (45.1)	23.2 (5.7)	208 (52.7)		23.8 (5.4)	−1.10 (366.29)		.27	
Help-seeking propensity (n=386)	178 (45.1)	25.8 (5.2)	208 (52.7)		25.9 (5.9)	−0.05 (383.52)		.96	
Indifference to depression stigma (n=383)	177 (44.8)	23.9 (6.9)	206 (52.2)		24.9 (6.3)	−1.45 (358.67)		.15	
IASMHS total (n=383)	177 (44.8)	72.9 (14.5)	206 (52.2)		74.5 (13.9)	−1.07 (367.30)		.28	

^a^“Undecided” responses were combined with “disagree” and “somewhat disagree” responses to form a “did not agree” category for those who did not indicate agreement.

^b^The total sample size <395 and percentages may not sum to 100% owing to item missingness.

^c^ Italicized *P* value denotes statistical significance.

**Table 6:** The Patient Health Questionnaire 9-item scale (PHQ-9) and Inventory of Attitudes Toward Seeking Mental Health Services (IASMHS) factor scores by agreement with using voice call to communicate with a professional to receive help for managing depression^a^.

Measure^b^	Agree			Did not agree			*t* test (*df*)		*P* value
	Values, n (%)	Values, mean (SD)	Values, n (%)		Values, mean (SD)				
PHQ-9 (n=385)	273 (69.1)	5.8 (5.5)	112 (28.4)		5.7 (5.6)	0.16 (204.16)		.87	
Psychological openness (n=388)	276 (69.9)	23.6 (5.6)	112 (28.4)		23.3 (5.3)	0.59 (217.47)		.56	
Help-seeking propensity (n=388)	276 (69.9)	26.5 (4.9)	112 (28.4)		24.3 (6.6)	3.24 (163.38)		*.001* ^c^	
Indifference to depression stigma (n=385)	275 (69.6)	24.8 (6.3)	110 (27.8)		23.6 (7.2)	1.54 (179.86)		.13	
IASMHS total (n=385)	275 (69.6)	74.9 (13.5)	110 (27.8)		70.9 (15.5)	2.38 (178.29)		*.02*	

^a^“Undecided” responses were combined with “disagree” and “somewhat disagree” responses to form a “did not agree” category for those who did not indicate agreement.

^b^The total sample size <395 and percentages may not sum to 100% owing to item missingness.

^c^ Italicized *P* value denotes statistical significance.

**Table 7:** The Patient Health Questionnaire 9-item scale (PHQ-9) and Inventory of Attitudes Toward Seeking Mental Health Services (IASMHS) factor scores by agreement with using mobile app to communicate with a professional to receive help for managing depressiona.

Measure^b^	Agree		Did not agree			*t* test (*df*)		*P* value
	Values, n (%)	Values, mean (SD)	Values, n (%)	Values, mean (SD)				
PHQ-9 (n=382)	176 (44.6)	7.0 (6.3)	206 (52.2)	4.8 (4.6)	3.89 (316.21)		*<.001* ^c^	
Psychological openness (n=384)	178 (45.1)	23.1 (5.9)	206 (52.2)	23.8 (5.2)	−1.23 (354.63)		.22	
Help-seeking propensity (n=384)	178 (45.1)	25.9 (5.6)	206 (52.2)	25.8 (5.6)	0.05 (374.41)		.96	
Indifference to depression stigma (n=381)	178 (45.1)	24.4 (6.7)	203 (51.4)	24.4 (6.5)	−0.09 (370.12)		.93	
IASMHS total (n=381)	178 (45.1)	73.3 (14.7)	203 (51.4)	73.9 (13.8)	−0.42 (365.43)		.67	

^a^“Undecided” responses were combined with “disagree” and “somewhat disagree” responses to form a “did not agree” category for those who did not indicate agreement.

^b^The total sample size <395 and percentages may not sum to 100% owing to item missingness.

^c^ Italicized *P* value denotes statistical significance.

**Table 8:** The Patient Health Questionnaire 9-item scale (PHQ-9) and Inventory of Attitudes Toward Seeking Mental Health Services (IASMHS) factor scores by agreement with using video call to communicate with a professional to receive help for managing depressiona.

Measure^b^	Agree			Did not agree			*t* test (*df*)		*P* value
	Values, n (%)	Values, mean (SD)	Values, n (%)		Values, mean (SD)				
PHQ-9 (n=384)	252 (63.8)	6.0 (5.6)	132 (33.4)		5.3 (5.4)	1.17 (275.01)		.24	
Psychological openness (n=386)	253 (64.1)	24.1 (5.5)	133 (33.7)		22.5 (5.6)	2.68 (264.83)		*.008* ^c^	
Help-seeking propensity (n=386)	253 (64.1)	26.3 (5.2)	133 (33.7)		25.1 (6.1)	1.95 (237.46)		.053	
Indifference to depression stigma (n=383)	251 (63.5)	25.0 (6.2)	132 (33.4)		23.4 (7.2)	2.19 (232.42)		*.03*	
IASMHS total (n=383)	251 (63.5)	75.2 (13.3)	132 (33.4)		70.8 (15.4)	2.81 (235.41)		*.005*	

^a^“Undecided” responses were combined with “disagree” and “somewhat disagree” responses to form a “did not agree” category for those who did not indicate agreement.

^b^The total sample size <395 and percentages may not sum to 100% owing to item missingness.

^c^ Italicized *P* value denotes statistical significance.

The results of the multivariable logistic regression analysis showed no statistically significant ORs (all *P*>.05) between respondents who agreed to use SMS text messaging to communicate with a professional to receive help for managing depression and education level, household income, health insurance, PHQ-9, psychological openness, help-seeking propensity, indifference to depression stigma, past mental health service use, unmet mental health need, or region (Multimedia Appendix 2).

Statistically significant ORs were found between respondents who agreed to use voice call to communicate with a professional to receive help for managing depression and help-seeking propensity, both as a dichotomy (score 17-32 vs score 0-16; adjusted OR 3.71, 95% CI 1.60-8.65; *P*=.002) and as a continuous linear effect (adjusted OR 1.10 per 1 unit increase, 95% CI 1.05-1.15; *P*<.001). An association was also found with the continuous version of indifference to depression stigma (adjusted OR 1.04 per 1 unit increase, 95% CI 1.00-1.08; *P*=.04). No statistically significant ORs (all *P*>.05) were found between respondents who agreed to use voice call to communicate with a professional to receive help for managing depression and education level, household income, health insurance, PHQ-9, psychological openness, indifference to depression stigma, past mental health service use, or unmet mental health need and region (Multimedia Appendix 3).

Statistically significant ORs were found between respondents who agreed to use mobile app to communicate with a professional to receive help for managing depression and PHQ-9, both as a dichotomy (score 10-27 vs score 0-9; adjusted OR 2.34, 95% CI 1.23-4.45; *P*=.01) and as a continuous linear effect (adjusted OR 1.06 per 1 unit increase, 95% CI 1.01-1.21; *P*=.02). No statistically significant associations (all *P*>.05) were found among respondents who agreed to use mobile app to communicate with a professional to receive help for managing depression and education level, household income, health insurance, psychological openness, help-seeking propensity, indifference to depression stigma, past mental health service use, unmet mental health need, or region (Multimedia Appendix 4).

Statistically significant associations were found between respondents who agreed to use video call to communicate with a professional to receive help for managing depression and help-seeking propensity as a dichotomy (score 17-27 vs score 0-16; adjusted OR 2.67, 95% CI 1.15-6.20; *P*=.02) as well as a continuous linear effect (adjusted OR 1.07 per 1 unit increase, 95% CI 1.03-1.12; *P*=.001). This was also true for indifference to depression stigma as a dichotomy (score 17-27 vs score 0-16; adjusted OR 2.35, 95% CI 1.22-4.51; *P*=.01) and as a continuous linear effect (adjusted OR 1.06 per 1 unit increase, 95% CI 1.03-1.10; *P*<.001). However, only a continuous linear effect was found for psychological openness (adjusted OR 1.06 per 1 unit increase, 95% CI 1.02-1.11; *P*=.002). No statistically significant ORs (all *P*>.05) were found between the respondents who agreed to use video call to communicate with a professional to receive help for managing depression and education level, household income, health insurance, PHQ-9, psychological openness, past mental health service use, unmet mental health need, or region (Multimedia Appendix 5).

A statistically significant interaction was found between depression severity using a cutoff score of 7 and depression stigma for respondent’s agreement to use SMS text messaging (score 0-16 vs 17-32 at a PHQ-9 score 7-27; adjusted OR 3.95, 95% CI 1.34-11.67, *P*=.02) or a mobile app (score 0-16 vs 17-32 at a PHQ-9 score 0-6, adjusted OR 0.23, 95% CI 0.80-0.67; and score 0-16 vs 17-32 at a PHQ-9 score 7-27, adjusted OR 3.19, 95% CI 1.07-9.50, *P*<.001). A statistically significant interaction was found between depression severity using a cutoff score of 10 and psychological openness for the respondent’s agreement to use video call (score 0-16 vs 17-32 at a PHQ-9 score 10-27; adjusted OR 0.19, 95% CI 0.05-0.68, *P*=.04). No significant interactions were found between depression severity using a cutoff score of 7 and psychological openness or help-seeking propensity. No significant interactions were found between depression severity using a cutoff score of 10 and depression stigma or help-seeking propensity (Multimedia Appendices 6 and 7).

### Qualitative Results

More than half (219/395, 55.4%) of the respondents indicated having concerns about using SMS text messaging to communicate with a professional about their mental health. The results of the study showed that 41.3% (163/395) of respondents selected “Yes” to the question, “Do you have any concerns about using a voice call to receive mental health treatment or counseling?” Furthermore, 32.7% (129/395) selected “Yes” when asked about video call, and 61% (241/395) selected “Yes” when asked about mobile app.

The concerns for each modality were coded into themes. With regard to SMS text messaging, 232 responses were coded and revealed the following concerns: privacy and confidentiality (77/232, 33.2%), communication issues (59/232, 25.4%), the impersonal feeling of communicating by SMS text messaging (54/232, 23.3%), does not like texting for this purpose (21/232, 9.1%), belief that the mode is insufficient for treatment (16/232, 6.9%), and does not send texts often or does not like to text (5/232, 2.2%):

Fear that message will go to wrong person; have experienced this.

Being able to not “read” into the text messages and not being able to understand “tone” and “cues” in text messages. Often times, things can be misunderstood or misconstrued.

Will this treatment be less personal? I like that, there is a low barrier to access but worry about the quality of treatment.

For voice call, 186 responses were coded and showed the following concerns: privacy and confidentiality (56/186, 30.1%), the impersonal feeling of communicating by voice call (54/186, 29%), communication issues (39/186, 21%), the belief that the mode is insufficient for treatment (28/186, 15.1%), does not like talking on the phone (4/186, 2.2%), connectivity issues (3/186, 1.6%), and other concerns such as time constraints or distractions (2/186, 0.5%):

Who’s listening besides the two of us?

It’s impersonal. You can’t read facial expressions. There’s also more concern for recording conversations.

The call may drop while I am talking.

With regard to video call, 119 responses were coded and revealed the following concerns: privacy and confidentiality (42/119, 35.3%), the belief that the mode is insufficient for treatment (26/119, 21.9%), the impersonal feeling of communicating by video call (21/119, 17.7%), technology issues (10/119, 8.4%), does not use video calling often or does not like video calling (7/119, 5.9%), concerns about being on camera (5/119, 4.2%), communication issues (4/119, 3.4%), and other concerns such as a lack of focus or not knowing how to use the technology (4/119, 3.4%):

They would actually be able to see me and videotape to use for future use.

While seeming as a better over the phone option, sometimes being able to remove yourself from your usual environment to have these conversations is a help in itself.

Technology is great when it works, but having difficulties when someone is in a fragile state could exacerbate the issue or delay treatment.

For mobile app, 251 responses were coded and showed the following concerns: privacy and confidentiality (84/251, 33.5%), the impersonal feel of communicating by mobile app (58/251, 23.1%), the belief that the mode is insufficient for treatment (52/251, 20.7%), technology issues (17/251, 6.8%), communication issues (15/251, 6%), does not use mobile app often or does not like using mobile app (10/251, 4%), unfamiliarity with the process (7/251, 2.8%), and other concerns such as cost and availability of mental health professionals to provide support (6/251, 2.4%):

I am unsure of how security would be maintained through an app. It also seems less personable than mental health treatment should be.

Can’t get a feel for the person providing the service.

App crashing, upgrades to app, and phone issues.

## Discussion

### Principal Findings

The findings of the study revealed a similar prevalence of depression among survey respondents (117/395, 29.6%) compared with that reported among non-Hispanic Black women in a large national survey (27%) [2]. Moreover, the results showed that younger Black American women were more likely to have higher levels of depression than their older counterparts. The 25 to 34 years age group, followed by the 18 to 24 years age group had the highest percentage of individuals with moderate, moderately severe, or severe depression compared with the older age groups. The results showed a trend of depression severity gradually decreasing among the older age groups. For example, the 35 to 44 years age group had lower levels of depression than the under 35 years age group and the 45 to 54 years age group had lower levels of depression than the 35 to 44 years age group. This finding is consistent with prior literature that reported that younger Black women (<50 years) had a higher prevalence of lifetime mood disorders (eg, major depressive disorder) than older Black women [1].

The results also revealed that Black American women have favorable views toward seeking mental health services comparable with non–Black American women [44]. Higher scores indicate more positive attitudes toward seeking professional psychological help (ranging from 0 to 32). Respondents’ reports of psychological openness (mean 23.53, SD 5.52) and help-seeking propensity (mean 25.91, SD 5.53) were comparable with both the respondents’ scores for psychological openness (mean 23.95, SD 4.53) and help-seeking propensity (mean 26.11, SD 4.89) from the preliminary study [36] and the adult female normative scores for psychological openness (mean 23.19, SD 6.00) and help-seeking propensity (mean 24.95, SD 4.74) [44]. The respondents’ scores on indifference to depression stigma (mean 24.50, SD 6.56) were similar to respondents’ scores for indifference to depression stigma (mean 23.58, SD 6.43) from the preliminary study [36].

A previous study found that African American women have less favorable attitudes toward professional help seeking than their non–African American counterparts [11]. However, the differences in the reported results between the studies may be due to significant differences in age and education level between the study respondents. The mean age of the women in the previous study was 20.9 years, and most participants (92.6%) reported attending a 4-year university program [11]. In comparison, the mean age of the women in our study was 44.8 (SD 18.4) years, and most of the respondents (312/395, 79%) had at least a bachelor’s degree. Therefore, the 24-year difference in the mean age between the study samples and the difference in education level could have contributed to the contrasting findings. Furthermore, the period between the occurrence of both studies may also explain the difference in attitudes toward seeking mental health services. The use of mental health services may be less stigmatizing currently than it was in the past. A recent study emphasized the significance of psychological experiences and self-critical symptoms (ie, indifference to depression stigma and psychological openness) in providing culturally sensitive mental health services to Black women [45].

Respondents were most comfortable with the use of voice call or video call to communicate with a professional to receive help for managing depression. The results supported the findings of the preliminary study that showed that Black American women were more comfortable with the use of video call than SMS text messaging to communicate with a professional to receive help for managing depression [34,36]. Furthermore, most respondents agreed that having the option to use voice call or video call to communicate with a professional if they are dealing with depression would be helpful. However, ≤50% of the respondents agreed that having the option to use a mobile app or SMS text messaging to communicate with a professional if they are dealing with depression would be helpful.

Younger Black American women (≤50 years) were significantly more likely to endorse the use of SMS text messaging, voice call, mobile app, and video call to communicate with a professional to receive help for managing depression. The results are consistent with the findings from the preliminary study and other published literature; in general, there is greater acceptance of the use of technology among younger adults, as older adults generally have many concerns [34,46]. Significant findings emerged regarding education. Respondents who reported having less than a bachelor’s degree were more likely to endorse the use of SMS text messaging or mobile app to communicate with a professional to receive help for managing depression than respondents with a bachelor’s degree or higher. This finding may largely be due to age as undergraduates are primarily between the ages of 18 and 24 years, the adult age group that uses SMS text messaging the most. Young adults are the most avid texters, with mobile phone users between the ages of 18 and 24 years exchanging an average of 109.5 messages per day compared with an average of 41.5 SMS text messages exchanged daily by the adult population as a whole [47].

Furthermore, respondents who agreed with the use of SMS text messaging to communicate with a professional to receive help for managing depression, as a group, were significantly more depressed than the group that did not indicate agreement with the use of SMS text messaging. Similarly, respondents who agreed to the use of a mobile app to communicate with a professional to receive help for managing depression, as a group, were significantly more depressed than the group that did not indicate agreement with the use of a mobile app. These findings may be primarily because younger African American women were more likely to have higher levels of depression and more likely to endorse the use of SMS text messaging or a mobile app. Respondents who agreed with the use of voice call to communicate with a professional to receive help for managing depression, as a group, had greater help-seeking propensity (ie, they were more likely to believe they are willing and able to seek professional psychological help) and more favorable attitudes toward seeking mental health services than the group who did not indicate agreement with the use of voice call. Respondents who agreed with the use of video call to communicate with a professional to receive help to manage depression, as a group, had greater psychological openness (ie, they were more open to acknowledging psychological problems and to the possibility of seeking professional help for them), greater indifference to depression stigma (ie, they were less concerned about what others might think should they find out that they were seeking professional help for psychological problems), and more favorable attitudes toward seeking mental health services than the group who did agree to use video call.

No statistically significant ORs were found among education level, household income, health insurance, past mental health service use, unmet mental health, or region and respondents who agreed to use any modality to communicate with a professional to receive support for managing depression. Psychological openness was significantly associated with respondents who agreed to use SMS text messaging to communicate with a professional to receive support to manage depression, although only as a continuous linear effect. In addition, no statistically significant ORs were associated with respondents who agreed to use SMS text messaging to communicate with a professional to receive support for managing depression.

The results revealed that higher help-seeking propensity scores (vs lower scores) increased the odds of agreement to use voice call and video call to communicate with a professional to receive support for managing depression, by nearly 2 to 3 times the odds. Moderate to severe depression severity (vs minimal to mild) increased the odds of using mobile app to communicate with a professional to receive support to manage depression by twice the odds. No statistically significant OR was found between depression severity and respondents’ agreement to use SMS text messaging, voice call, or video call.

Higher scores for indifference to depression stigma (high vs low scores) increased the odds of indicating agreement to using video call to communicate with a professional to receive support for managing depression, by 1.35 times the odds. This effect was also seen for the continuous linear effect. This finding may indicate that indifference to depression stigma makes one more open to more visible modes of communication to receive support for managing depression (eg, video call). No significant ORs were found between help-seeking propensity (higher vs lower scores) and respondents who agreed to use a mobile app or SMS text messaging. Similarly, no statistically significant OR was found between depression severity and indifference to depression stigma and respondents who agreed to use SMS text messaging, voice call, or video call.

Privacy and confidentiality, communication issues (eg, misinterpreting text), and the impersonal feel of communicating by mobile phone (via SMS text messaging, voice call, mobile app, or video call) were the primary concerns for some respondents. In addition, the belief that the use of the modalities may be insufficient for treatment in some situations and possible technology issues (eg, disconnection or poor connectivity) disrupting communication were also concerns. This presents a challenge to the adoption and sustained use of these modalities to support Black women in managing depression. However, efforts should be made to eliminate or mitigate barriers to the use of telehealth modalities, such as (1) providing clear communication about what is being done to protect individuals’ privacy and confidentiality; (2) simplifying SMS text messages to avoid confusion; (3) personalizing automated interactions and providing options for individuals to connect with a human, if desired; and (4) screening to determine best or preferred modalities for providing treatment, support, information, and resources—this includes screening for access to an adequate broadband internet connection [48].

### Strengths and Limitations

The main strengths of the cross-sectional survey study were the large sample size and wide age distribution—the study had an adequate sample size to produce sufficient precision and was representative of age groups of Black women in the United States. A total of 395 respondents completed the survey through email and social media recruitment. However, the main limitations are the mode used to administer the survey and the recruitment methods. Owing to the sensitive nature of the questions and for convenience, the computer-assisted web interviewing data collection technique was used to administer the survey. Although this method may increase privacy and reduce respondent burden in completing the survey, women who do not have email access or a social media account may not be able to complete the survey by accessing the link. Furthermore, mobile phone ownership of the respondents in this study was higher than the percentage reported in the general population (99% vs 80%, respectively). In general, individuals with greater access to technology are more likely to have higher incomes and greater help-seeking propensity. They are also more likely to participate in the study; although this may limit the generalizability of the results, we accounted for lower technology options, such as voice call and SMS text messaging.

With regard to recruitment, respondents were recruited through convenience sampling and encouraged to share the survey email or social media posts with their networks. Although no personally identifiable information was collected in the survey and respondents accessed the survey through an anonymous link, social desirability bias could have resulted if the respondent personally knew the principal investigator (TM). In addition, the sample consisted mostly of highly educated women (312/395, 79% had at least a bachelor’s degree) with health insurance (371/395, 93.9% indicated that they had health insurance) and relatively higher incomes (232/395, 58.7% reported an income of ≥US $50,000). In addition, considerations for dichotomizing the age and education variables are that it may mask differences between age groups (eg, 18-24 vs 35-44 years) and education level (eg, high school diploma or General Educational Development tests vs some college, ≤4-year degree). However, age was dichotomized into 2 groups (≤50 years and ≥50 years) and education was categorized into 2 levels (less than a bachelor’s degree and a bachelor’s degree or higher) to be consistent with the preliminary study and to allow for the comparison of results [34,36].

As this was not a random sample, our ability to broadly generalize the findings to all Black American women was limited. Furthermore, the grouping of responses potentially resulted in limited sample sizes for some of the variables we explored. Black American women with health insurance are still less likely to use mental health services compared with their White counterparts [49]. With regard to our use of the PHQ-9 to assess the presence and severity of depression, the instrument may be limited in its ability to identify the complete range of relevant depressive symptoms among Black women. Black women who have symptoms of depression were found to report sleep disturbances, self-criticism, and irritability more often than commonly assessed symptoms such as depressed mood [45].

### Conclusions and Future Directions

This study was conducted before the COVID-19 pandemic. The findings showed that Black American women, in general, have favorable views toward seeking mental health services and were most comfortable with the use of voice call or video call to communicate with a professional to receive help for managing depression. In addition, younger age groups (<50 years) were more likely to endorse the use of SMS text messaging and mobile apps to receive support for managing depression. Owing to the rapid uptake of digital health tools during the COVID-19 pandemic, we hypothesize that the acceptability of using mobile technology for mental health support has increased among Black women. However, future engagement strategies should seek to provide information, establish trust, and provide accessible and affordable culturally relevant options for mental health support. The preferences and appropriateness of modalities for providing support should be assessed on an individual basis. For example, a client may prefer SMS text messaging for quick check-ins; however, they would prefer a video call when a greater level of support is needed. This study’s findings can be used to inform public health interventions and the development and implementation of digital mental health technologies for minoritized groups, such as a mobile app to support the management of depression among Black American women [33,50]. Although attitudes toward the use of mobile technology to support the management of depression among Black American women, in general, are more favorable than in the past, for the benefits to be realized, we must move beyond identifying needs to continue to increase awareness of the importance of mental health care and provide more tangible support.

## Appendix

Multimedia Appendix 1 Attitudes toward seeking mental health services and the use of mobile technology survey.

Multimedia Appendix 2 Multivariable logistic regression models for attitudes toward using SMS text messaging to communicate with a professional to receive support for managing depression.

Multimedia Appendix 3 Multivariable logistic regression models for attitudes toward using voice call to communicate with a professional to receive support for managing depression.

Multimedia Appendix 4 Multivariable logistic regression models for attitudes toward using mobile apps to communicate with a professional to receive support for managing depression.

Multimedia Appendix 5 Multivariable logistic regression models for attitudes toward using video call to communicate with a professional to receive support for managing depression.

Multimedia Appendix 6 Significant interactions for a depression severity Patient Health Questionnaire 9-item scale cutoff score of 7 (0-6 vs 7-27) in multivariable logistic regression models for attitudes toward using each modality to communicate with a professional to receive support for managing depression.

Multimedia Appendix 7 Significant interactions for a depression severity Patient Health Questionnaire 9-item scale cutoff score of 10 (0-9 vs 10-27) in multivariable logistic regression models for attitudes toward using each modality to communicate with a professional to receive support for managing depression.

## Abbreviations

IASMHS: Inventory of Attitudes Toward Seeking Mental Health Services
mHealth: mobile health
OR: odds ratio
PHQ-9: Patient Health Questionnaire 9-item scale
TAM: Technology Acceptance Model
TPB: Theory of Planned Behavior

## Data Availability

The raw data supporting the conclusions of this paper will be made available by the authors without undue reservation.
