# Supplementary material for: Attitudes Toward Seeking Mental Health Services and Mobile Technology to Support the Management of Depression Among Black American Women: Cross-Sectional Survey Study
Source: J Med Internet Res. 2023 Jul 19;25:e45766. doi: 10.2196/45766 (PMC10398364; doi:10.2196/45766)
Supplement: Multimedia Appendix 2 [file jmir_v25i1e45766_app2.docx]

**Multimedia Appendix 2.** Multivariable logistic regression models for attitudes toward using *SMS* *text messaging* to communicate with a professional to receive support for managing *depression.*

|  | | Agree^a^, % (n/N) | Unadjusted OR^b^ (95% CI) | Age-adjusted^c^ OR (95% CI) | Multivariably adjusted^d^ OR (95% CI) | *P* value for pairwise comparison vs reference | Multivariably adjusted^d^ OR (95% CI) per 1 unit change | *P* value for continuous linear effect |
| --- | --- | --- | --- | --- | --- | --- | --- | --- |
| **Age range (years)** | | | | | | | N/A^e^ | N/A |
|  | 18-24 | 56 (33/59) | Reference^f^ | N/A | N/A | N/A |  |  |
|  | 25-34 | 42 (51/98) | 0.85 (0.45-1.64) | N/A | N/A | N/A |  |  |
|  | 35-44 | 63 (29/46) | 1.34 (0.61-2.96) | N/A | N/A | N/A |  |  |
|  | 45-54 | 43 (25/58) | 0.60 (0.29-1.24) | N/A | N/A | N/A |  |  |
|  | 55-64 | 22 (12/55) | *0.23 (0.10-0.53)*^g^ | N/A | N/A | N/A |  |  |
|  | ≥65 | 35 (28/79) | *0.49 (0.24-0.99)* | N/A | N/A | N/A |  |  |
| **Age group (years)** | | | | | | | N/A | N/A |
|  | <50 | 33 (53/163) | Reference | N/A | N/A | N/A |  |  |
|  | ≥50 | 54 (125/232) | *0.45 (0.29-0.68)* | N/A | N/A | N/A |  |  |
| **Education** | | | | | | | N/A | N/A |
|  | Less than bachelor’s degree | 42.3 (132/312) | Reference | Reference | Reference | Reference |  |  |
|  | Bachelor’s degree or higher | 55.4 (46/83) | *0.58 (0.35-0.95)* | 0.63 (0.38-1.03) | 0.61 (0.36-1.02) | .06 |  |  |
| **Household income ($)** | | | | | | | N/A | N/A |
|  | <25,000 | 46.3 (31/67) | Reference^h^ | Reference^i^ | Reference^j^ | Reference |  |  |
|  | 25,000-49,999 | 57.6 (53/92) | 1.62 (0.86-3.06) | *2.18 (1.12-4.27)* | *2.17 (1.09-4.32)* | *.03* |  |  |
|  | 50,000-100,000 | 42 (58/138) | 0.92 (0.51-1.67) | 1.46 (0.75-2.82) | 1.58 (0.80-3.13) | .19 |  |  |
|  | >100,000 | 38.3 (36/94) | 0.72 (0.38-1.36) | 1.14 (0.57-2.29) | 1.23 (0.60-2.53) | .57 |  |  |
| **Health insurance** | | | | | | | N/A | N/A |
|  | Yes | 43.7 (162/371) | 0.43 (0.18-1.04) | 0.53 (0.22-1.30) | 0.51 (0.20-1.32) | .17 |  |  |
|  | No | 65.2 (15/23) | Reference | Reference | Reference | Reference |  |  |
| **Depression severity (PHQ-9^k^ score)^l^** | | | | | | | 1.04 (0.99-1.10) | .09 |
|  | 0-9 | 41.8 (131/313) | Reference | Reference | Reference | Reference |  |  |
|  | 10-27 | 57.7 (45/78) | *1.82 (1.10-3.01)* | 1.38 (0.81-2.35) | 1.56 (0.84-2.89) | .16 |  |  |
| **Psychological openness^m^ (score)** | | | | | | | 0.10 (0.96-1.04) | .84 |
|  | 0-16 | 52.1 (25/48) | Reference | Reference | Reference | Reference |  |  |
|  | 17-32 | 44.2 (153/346) | 0.76 (0.42-1.39) | 0.89 (0.48-1.65) | 0.93 (0.49-1.75) | .82 |  |  |
| **Help-seeking propensity^m^ (score)** | | | | | | | 1.03 (0.99-1.08) | .13 |
|  | 0-16 | 46.4 (13/28 ) | Reference | Reference | Reference | Reference |  |  |
|  | 17-32 | 45.1 (165/366) | 0.99 (0.46-2.13) | 1.32 (0.59-2.93) | 1.65 (0.70-3.87) | .25 |  |  |
| **Indifference to depression stigma^m^ (score)** | | | | | | | 0.10 (0.96-1.03) | .75 |
|  | 0-16 | 58.3 (28/48) | Reference | Reference | Reference | Reference |  |  |
|  | 17-32 | 43.4 (149/343) | 0.57 (0.31-1.06) | 0.71 (0.38-1.34) | 0.67 (0.35-1.27) | .21 |  |  |
| **Past mental health service use** | | | | | | | N/A | N/A |
|  | Yes | 49.3 (73/148) | 1.32 (0.87-1.99) | 1.00 (0.64-1.57) | 0.99 (0.59-1.65) | .96 |  |  |
|  | No | 41.7 (101/242) | Reference | Reference | Reference | Reference |  |  |
| **Unmet mental health need** | | | | | | | N/A | N/A |
|  | Yes | 49.7 (79/159) | 1.34 (0.88-2.02) | 0.85 (0.52-1.38) | 0.82 (0.48-1.37) | .44 |  |  |
|  | No | 57.2 (91/217) | Reference | Reference | Reference | Reference |  |  |
| **Region** | | | | | | | N/A | N/A |
|  | Midwest | 36.7 (22/60) | 0.63 (0.35-1.13) | 0.61 (0.34-1.11) | 0.65 (0.35-1.18) | .16 |  |  |
|  | Northeast | 36.2 (25/69) | 0.64 (0.37-1.13) | 0.60 (0.34-1.07) | 0.59 (0.33-1.07) | .08 |  |  |
|  | West | 52.9 (18/34) | 1.27 (0.61-2.63) | 1.39 (0.65-2.94) | 1.46 (0.67-3.18) | .34 |  |  |
|  | South | 48 (110/229) | Reference^n^ | Reference^o^ | Reference^p^ | Reference |  |  |

^a^Agree indicates agreement with the use of SMS text messaging to communicate with a professional to receive support for managing depression.

^b^OR: odds ratio.

^c^Adjusted for age only.

^d^Adjusted for age and history of depression.

^e^N/A: not applicable.

^f^Overall test of effect, *df*=5, *P*<.001.

^g^Italicized odds ratios (OR) denotes statistical significance.

^h^Overall test of effect, *df*=3, *P*=.05.

^i^Overall test of effect, *df*=3, *P*=.11.

^j^Overall test of effect, *df*=3, *P*=.11.

^k^PHQ-9: Patient Health Questionnaire 9-item scale.

^l^A score of ≥10 on the PHQ-9 indicates at least moderate depression severity.

^m^Higher scores indicate more positive attitudes toward seeking professional psychological help.

^n^Overall test of effect, *df* =3, *P*=.16.

^o^Overall test of effect, *df*=3, *P*=.09.

^p^Overall test of effect, *df*=3, *P*=.11.
