# Supplementary material for: Attitudes Toward Seeking Mental Health Services and Mobile Technology to Support the Management of Depression Among Black American Women: Cross-Sectional Survey Study
Source: J Med Internet Res. 2023 Jul 19;25:e45766. doi: 10.2196/45766 (PMC10398364; doi:10.2196/45766)
Supplement: Multimedia Appendix 3 [file jmir_v25i1e45766_app3.docx]

**Multimedia Appendix 3.** Multivariable logistic regression models for attitudes toward using *voice call* to communicate with a professional to receive support for managing *depression.*

|  | | Agree^a^, % (n/N) | Unadjusted OR^b^ (95% CI) | Age-adjusted^c^ OR (95% CI) | Multivariably adjusted^d^ OR (95% CI) | *P* value for pairwise comparison vs reference | Multivariably adjusted^d^ OR (95% CI) per 1 unit change | *P* value for continuous linear effect |
| --- | --- | --- | --- | --- | --- | --- | --- | --- |
| **Age range (years)** | | | | | | | N/A^e^ | N/A |
|  | 18-24 | 73 (43/59) | Reference^f^ | N/A | N/A | N/A |  |  |
|  | 25-34 | 67 (66/98) | 0.77 (0.38-1.57) | N/A | N/A | N/A |  |  |
|  | 35-44 | 87 (40/46) | 2.48 (0.88-6.96) | N/A | N/A | N/A |  |  |
|  | 45-54 | 79 (46/58) | 1.43 (0.61-3.36) | N/A | N/A | N/A |  |  |
|  | 55-64 | 56 (31/55) | 0.50 (0.23-1.10) | N/A | N/A | N/A |  |  |
|  | ≥65 | 63 (50/79) | 0.78 (0.37-1.64) | N/A | N/A | N/A |  |  |
| **Age group (years)** | | | | | | | N/A | N/A |
|  | <50 | 63 (102/163) | Reference | N/A | N/A | N/A |  |  |
|  | ≥50 | 75 (174/232) | *0.62 (0.40-0.96)*^g^ | N/A | N/A | N/A |  |  |
| **Education** | | | | | | | N/A | N/A |
|  | Less than bachelor’s degree | 69.9 (218/312) | Reference | Reference | Reference | Reference |  |  |
|  | Bachelor’s degree or higher | 69.9 (58/83) | 0.96 (0.56-1.65) | 0.99 (0.57-1.70) | 1.02 (0.59-1.77) | .95 |  |  |
| **Household income ($)** | | | | | | | N/A | N/A |
|  | <25,000 | 65.7 (44/67) | Reference^h^ | Reference^i^ | Reference^j^ | Reference |  |  |
|  | 25,000-49,999 | 75 (69/92) | 1.64 (0.81-3.33) | 1.79 (0.86-3.73) | 1.86 (0.89-3.91) | .10 |  |  |
|  | 50,000-100,000 | 68.1 (94/138) | 1.12 (0.60-2.10) | 1.30 (0.64-2.61) | 1.48 (0.72-3.03) | .28 |  |  |
|  | >100,000 | 72.3 (68/94) | 1.36 (0.68-2.70) | 1.56 (0.74-3.30) | 1.78 (0.83-3.82) | .14 |  |  |
| **Health insurance** | | | | | | | N/A | N/A |
|  | Yes | 69.3 (257/371) | 0.66 (0.24-1.83) | 0.70 (0.25-1.96) | 0.83 (0.29-2.38) | .74 |  |  |
|  | No | 78.3 (18/23) | Reference | Reference | Reference | Reference |  |  |
| **Depression severity (PHQ-9^k^ score)^l^** | | | | | | | 0.98 (0.93-1.03) | .40 |
|  | 0-9 | 69.3 (217/313) | Reference | Reference | Reference | Reference |  |  |
|  | 0-27 | 71.8 (56/78) | 1.12 (0.64-1.95) | 1.02 (0.56-1.86) | 0.94 (0.48-1.85) | .86 |  |  |
| **Psychological openness^m^ (score)** | | | | | | | 1.02 (0.98-1.06) | .47 |
|  | 0-16 | 75 (36/48) | Reference | Reference | Reference | Reference |  |  |
|  | 17-32 | 69.4 (240/346) | 0.80 (0.40-1.60) | 0.84 (0.42-1.68) | 0.86 (0.42-1.73) | .67 |  |  |
| **Help-seeking propensity^m^ (score)** | | | | | | | *1.10 (1.05-1.15)* | *<.0001* |
|  | 0-16 | 50 (14/28) | Reference | Reference | Reference | Reference |  |  |
|  | 17-32 | 71.6 (262/366) | *2.67 (1.23-5.81)* | *3.05 (1.37-6.79)* | *3.71 (1.60-8.65)* | *.002* |  |  |
| **Indifference to depression stigma^m^ (score)** | | | | | | | *1.04 (1.00-1.08)* | *.04* |
|  | 0-16 | 68.8 (33/48) | Reference | Reference | Reference | Reference |  |  |
|  | 17-32 | 70.6 (242/343) | 1.16 (0.60-2.23) | 1.23 (0.65-2.48) | 1.30 (0.66-2.57) | .44 |  |  |
| **Past mental health service use** | | | | | | | N/A | N/A |
|  | Yes | 73 (108/148) | 1.20 (0.76-1.90) | 1.14 (0.70-1.86) | 1.13 (0.65-1.97) | .67 |  |  |
|  | No | 67.8 (164/242) | Reference | Reference | Reference | Reference |  |  |
| **Unmet mental health need** | | | | | | | N/A | N/A |
|  | Yes | 74.2 (118/159) | 1.45 (0.91-2.30) | 1.42 (0.83-2.41) | 1.22 (0.70-2.15) | .48 |  |  |
|  | No | 90.6 (144/217) | Reference | Reference | Reference | Reference |  |  |
| **Region** | | | | | | | N/A | N/A |
|  | Midwest | 66.7 (40/60) | 0.69 (0.37-1.29) | 0.69 (0.37-1.29) | 0.69 (0.37-1.29) | .25 |  |  |
|  | Northeast | 60.9 (42/69) | *0.51 (0.29-0.91)* | *0.51 (0.29-0.90)* | *0.52 (0.29-0.93)* | *.03* |  |  |
|  | West | 61.8 (21/34) | 0.63 (0.29-1.39) | 0.64 (0.29-1.41) | 0.68 (0.30-1.55) | .36 |  |  |
|  | South | 74.2 (170/229) | Reference^n^ | Reference^o^ | Reference^p^ | Reference |  |  |

^a^Agree indicates agreement with the use of voice call to communicate with a professional to receive support for managing depression.

^b^OR: odds ratio.

^c^Adjusted for age only.

^d^Adjusted for age and history of depression.

^e^N/A: not applicable.

^f^Overall test of effect, *df*=5, *P*=.03.

^g^Italicized odds ratios (OR) denotes statistical significance.

^h^Overall test of effect, *df*=3, *P*=.49.

^i^Overall test of effect, *df*=3, *P*=.42.

^j^Overall test of effect, *df*=3, *P*=.36.

^k^PHQ-9: Patient Health Questionnaire 9-item scale.

^l^A score of ≥10 on the PHQ-9 indicates at least moderate depression severity.

^m^Higher scores indicate more positive attitudes toward seeking professional psychological help.

^n^Overall test of effect, *df*=3, *P*=.11.

^o^Overall test of effect, *df*=3, *P*=.11.

^p^Overall test of effect, *df*=3, *P*=.14.
