# Supplementary material for: Attitudes Toward Seeking Mental Health Services and Mobile Technology to Support the Management of Depression Among Black American Women: Cross-Sectional Survey Study
Source: J Med Internet Res. 2023 Jul 19;25:e45766. doi: 10.2196/45766 (PMC10398364; doi:10.2196/45766)
Supplement: Multimedia Appendix 4 [file jmir_v25i1e45766_app4.docx]

**Multimedia Appendix 4.** Multivariable logistic regression models for attitudes toward using *mobile apps* to communicate with a professional to receive support for managing *depression.*

|  | | Agree^a^, % (n/N) | Unadjusted OR^b^ (95% CI) | Age-adjusted^c^ OR (95% CI) | Multivariably adjusted^d^ OR (95% CI) | *P* value for pairwise comparison versus reference | Multivariably adjusted^d^ OR (95% CI) per 1 unit change | *P* value for continuous linear effect |
| --- | --- | --- | --- | --- | --- | --- | --- | --- |
| **Age range (years)** | | | | | | | N/A^e^ | N/A |
|  | 18-24 | 59 (35/59) | Reference^f^ | N/A | N/A | N/A |  |  |
|  | 25-34 | 55 (54/98) | 0.84 (0.44-1.62) | N/A | N/A | N/A |  |  |
|  | 35-44 | 63 (29/46) | 1.17 (0.53-2.58) | N/A | N/A | N/A |  |  |
|  | 45-54 | 41 (24/58) | 0.48 (0.23-1.01) | N/A | N/A | N/A |  |  |
|  | 55-64 | 27 (15/55) | *0.26 (0.12-0.58)*^g^ | N/A | N/A | N/A |  |  |
|  | ≥65 | 27 (21/79) | *0.29 (0.14-0.61)* | N/A | N/A | N/A |  |  |
| **Age group (years)** | | | | | | | N/A | N/A |
|  | <50 | 29 (47/163) | Reference | N/A | N/A | N/A |  |  |
|  | ≥50 | 56 (131/232) | *0.34 (0.22-0.53)* | N/A | N/A | N/A |  |  |
| **Education** | | | | | | | N/A | N/A |
|  | Less than bachelor’s degree | 42.6 (133/312) | Reference | Reference | Reference | Reference |  |  |
|  | Bachelor’s degree or higher | 54.2 (45/83) | 0.60 (0.37-0.99) | 0.66 (0.40-1.11) | 0.60 (0.36-1.02) | .06 |  |  |
| **Household income ($)** | | | | | | | N/A | N/A |
|  | <25,000 | 46.3 (31/67) | Reference^h^ | Reference^i^ | Reference^j^ | Reference |  |  |
|  | 25,000-49,999 | 52.2 (48/92) | 1.25 (0.66-2.38) | 1.94 (0.98-3.84) | 1.76 (0.87-3.56) | .11 |  |  |
|  | 50,000-100,000 | 45.7 (63/138) | 0.99 (0.55-1.79) | 1.97 (0.99-3.90) | 1.91 (0.94-3.86) | .07 |  |  |
|  | >100,000 | 38.3 (36/94) | 0.69 (0.37-1.32) | 1.35 (0.66-2.76) | 1.27 (0.61-2.66) | .53 |  |  |
| **Health insurance** | | | | | | | N/A | N/A |
|  | Yes | 44.5 (165/371) | 0.77 (0.33-1.80) | 1.02 (0.43-2.42) | 0.83 (0.33-2.10) | .70 |  |  |
|  | No | 52.2 (12/23) | Reference | Reference | Reference | Reference |  |  |
| **Depression severity (PHQ-9^k^ score)^l^** | | | | | | | *1.06 (1.01-1.12)* | *.02* |
|  | 0-9 | 40.3 (126/313) | Reference | Reference | Reference | Reference |  |  |
|  | 10-27 | 64.1 (50/78) | *2.63 (1.56-4.43)* | *1.90 (1.09-3.30)* | *2.34 (1.23-4.45)* | *.01* |  |  |
| **Psychological openness^m^ (score)** | | | | | | | 0.99 (0.95-1.03) | .62 |
|  | 0-16 | 54.2 (26/48) | Reference | Reference | Reference | Reference |  |  |
|  | 17-32 | 43.9 (152/346) | 0.70 (0.38-1.28) | 0.84 (0.44-1.57) | 0.78 (0.41-1.47) | .44 |  |  |
| **Help-seeking propensity^m^ (score)** | | | | | | | 1.04 (0.997-1.08) | .07 |
|  | 0-16 | 53.6 (15/28) | Reference | Reference | Reference | Reference |  |  |
|  | 17-32 | 44.5 (163/366) | 0.73 (0.34-1.58) | 1.02 (0.46-2.28) | 1.02 (0.44-2.36) | .97 |  |  |
| **Indifference to depression stigma^m^ (score)** | | | | | | | 1.03 (.99-1.06) | .16 |
|  | 0-16 | 47.9 (23/48) | Reference | Reference | Reference | Reference |  |  |
|  | 17-32 | 45.2 (155/343) | 0.95 (0.52-1.74) | 1.27 (0.68-2.39) | 1.21 (0.63-2.30) | .57 |  |  |
| **Past mental health service use** | | | | | | | N/A | N/A |
|  | Yes | 49.3 (73/148) | 1.29 (0.85-1.95) | 0.89 (0.57-1.41) | 0.86 (0.51-1.44) | .55 |  |  |
|  | No | 41.7 (101/242) | Reference | Reference | Reference | Reference |  |  |
| **Unmet mental health need** | | | | | | | N/A | N/A |
|  | Yes | 56 (89/159) | 2.16 (1.42-3.29) | 1.44 (0.89-2.35) | 1.39 (0.83-2.34) | .21 |  |  |
|  | No | 50.3 (80/217) | Reference | Reference | Reference | Reference |  |  |
| **Region** | | | | | | | N/A | N/A |
|  | Midwest | 38.3 (23/60) | 0.68 (0.38-1.22) | 0.65 (0.36-1.19) | 0.65 (0.35-1.19) | .16 |  |  |
|  | Northeast | 37.7 (26/69) | 0.69 (0.40-1.21) | 0.64 (0.36-1.13) | 0.63 (0.35-1.13) | .12 |  |  |
|  | West | 47.1 (16/34) | 1.22 (0.57-2.61) | 1.26 (0.57-2.77) | 1.34 (0.59-3.02) | .49 |  |  |
|  | South | 48 (110/229) | Reference^n^ | Reference^o^ | Reference^p^ | Reference |  |  |

^a^Agree indicates agreement with the use of mobile app to communicate with a professional to receive support for managing depression.

^b^OR: odds ratio.

^c^Adjusted for age only.

^d^Adjusted for age and history of depression.

^e^N/A: not applicable.

^f^Overall test of effect, *df*=5, *P*<.0001.

^g^Italicized odds ratios (OR) denotes statistical significance.

^h^Overall test of effect, *df*=3, *P*=.26.

^i^Overall test of effect, *df*=3, *P*=.14.

^j^Overall test of effect, *df*=3, *P*=.20.

^k^PHQ-9: Patient Health Questionnaire 9-item scale.

^l^A score of ≥10 on the PHQ-9 indicates at least moderate depression severity.

^m^Higher scores indicate more positive attitudes toward seeking professional psychological help.

^n^Overall test of effect, *df*=3, *P*=.32.

^o^Overall test of effect, *df*=3, *P*=.21.

^p^Overall test of effect, *df*=3, *P*=.19.
