# Supplementary material for: Attitudes Toward Seeking Mental Health Services and Mobile Technology to Support the Management of Depression Among Black American Women: Cross-Sectional Survey Study
Source: J Med Internet Res. 2023 Jul 19;25:e45766. doi: 10.2196/45766 (PMC10398364; doi:10.2196/45766)
Supplement: Multimedia Appendix 5 [file jmir_v25i1e45766_app5.docx]

**Multimedia Appendix 5.** Multivariable logistic regression models for attitudes toward using *video call* to communicate with a professional to receive support for managing *depression.*

|  | | Agree^a^, % (n/N) | Unadjusted OR^b^ (95% CI) | Age-adjusted^c^ OR (95% CI) | Multivariably adjusted^d^ OR (95% CI) | *P* value for pairwise comparison versus reference | Multivariably adjusted^d^ OR (95% CI) per 1 unit change | *P* value for continuous linear effect |
| --- | --- | --- | --- | --- | --- | --- | --- | --- |
| **Age range (years)** | | | | | | | N/A^e^ | N/A |
|  | 18-24 | 71 (42/59) | Reference^f^ | N/A | N/A | N/A |  |  |
|  | 25-34 | 68 (67/98) | 0.88 (0.43-1.77) | N/A | N/A | N/A |  |  |
|  | 35-44 | 83 (38/46) | 1.92 (0.75-4.96) | N/A | N/A | N/A |  |  |
|  | 45-54 | 69 (40/58) | 0.90 (0.41-1.99) | N/A | N/A | N/A |  |  |
|  | 55-64 | 49 (27/55) | *0.42 (0.19-0.92)*^g^ | N/A | N/A | N/A |  |  |
|  | ≥65 | 51 (40/79) | 0.49 (0.24-1.02) | N/A | N/A | N/A |  |  |
| **Age group (years)** | | | | | | | N/A | N/A |
|  | <50 | 53 (86/163) | Reference | N/A | N/A | N/A |  |  |
|  | ≥50 | 72 (168/232) | *0.48 (0.31-0.73)* | N/A | N/A | N/A |  |  |
| **Education** | | | | | | | N/A | N/A |
|  | Less than bachelor’s degree | 64.7 (202/312) | Reference | Reference | Reference | Reference |  |  |
|  | Bachelor’s degree or higher | 62.7 (52/83) | 1.08 (0.65-1.81) | 1.17 (0.69-1.97) | 1.11 (0.65-1.88) | .71 |  |  |
| **Household income ($)** | | | | | | | N/A | N/A |
|  | <25,000 | 61.2 (41/67) | Reference^h^ | Reference^i^ | Reference^j^ | Reference |  |  |
|  | 25,000-49,999 | 67.4 (62/92) | 1.25 (0.64-2.44) | 1.72 (0.85-3.51) | 1.62 (0.79-3.35) | .19 |  |  |
|  | 50,000-100,000 | 60.1 (83/138) | 0.95 (0.52-1.76) | 1.57 (0.78-3.16) | 1.54 (0.75-3.16) | .24 |  |  |
|  | >100,000 | 71.3 (67/94) | 1.51 (0.77-2.97) | *2.44 (1.15-5.19)* | *2.37 (1.10-5.11)* | *.03* |  |  |
| **Health insurance** | | | | | | | N/A | N/A |
|  | Yes | 63.9 (237/371) | 0.82 (0.33-2.05) | 1.01 (0.40-2.55) | 0.92 (0.34-2.49) | .87 |  |  |
|  | No | 69.6 (16/23) | Reference | Reference | Reference | Reference |  |  |
| **Depression severity (PHQ-9^k^ score)^l^** | | | | | | | 0.98 (0.93-1.04) | .49 |
|  | 0-9 | 63.3 (198/313) | Reference | Reference | Reference | Reference |  |  |
|  | 10-27 | 69.2 (54/78) | 1.29 (0.75-2.22) | 0.98 (0.55-1.75) | 0.93 (0.48-1.80) | .83 |  |  |
| **Psychological openness^m^ (score)** | | | | | | | *1.06 (1.02-1.11)* | *.002* |
|  | 0-16 | 56.3 (27/48) | Reference | Reference | Reference | Reference |  |  |
|  | 17-32 | 65.3 (226/346) | 1.57 (0.85-2.90) | 1.82 (0.97-3.43) | 1.70 (0.90-3.24) | .10 |  |  |
| **Help-seeking propensity^m^ (score)** | | | | | | | *1.07 (1.03-1.121)* | *.001* |
|  | 0-16 | 50 (14/28) | Reference | Reference | Reference | Reference |  |  |
|  | 17-32 | 65.3 (239/366) | 2.01 (0.93-4.35) | *2.66 (1.19-5.94)* | *2.67 (1.15-6.20)* | *.02* |  |  |
| **Indifference to depression stigma^m^ (score)** | | | | | | | *1.06 (1.03-1.10)* | *<.001* |
|  | 0-16 | 52.1 (25/48) | Reference | Reference | Reference | Reference |  |  |
|  | 17-32 | 65.9 (226/343) | *1.91 (1.04-3.51)* | *2.49 (1.31-4.72)* | *2.35 (1.22-4.51)* | *.01* |  |  |
| **Past mental health service use** | | | | | | | N/A | N/A |
|  | Yes | 68.2 (101/148) | 1.26 (0.81-1.95) | 0.99 (0.62-1.60) | 0.91 (0.53-1.55) | .72 |  |  |
|  | No | 62 (150/242 ) | Reference | Reference | Reference | Reference |  |  |
| **Unmet mental health need** | | | | | | | N/A | N/A |
|  | Yes | 74.2 (118/159) | *2.25 (1.43-3.54)* | *1.90 (1.13-3.18)* | 1.65 (0.95-2.84) | .07 |  |  |
|  | No | 76.1 (121/217) | Reference | Reference | Reference | Reference |  |  |
| **Region** | | | | | | | N/A | N/A |
|  | Midwest | 58.3 (35/60) | 0.59 (0.33-1.06) | 0.57 (0.32-1.04) | 0.56 (0.30-1.01) | .06 |  |  |
|  | Northeast | 60.9 (42/69) | 0.70 (0.40-1.25) | 0.67 (0.38-1.20) | 0.63 (0.35-1.14) | .13 |  |  |
|  | West | 47.1 (16/34) | 0.48 (0.22-1.04) | 0.48 (0.22-1.04) | *0.42 (0.19-0.94)* | *.03* |  |  |
|  | South | 69.9 (160/229) | Reference^n^ | Reference^o^ | Reference^p^ | Reference |  |  |

^a^Agree indicates agreement with the use of video call to communicate with a professional to receive support for managing depression.

^b^OR: odds ratio.

^c^Adjusted for age only.

^d^Adjusted for age and history of depression.

^e^N/A: not applicable.

^f^Overall test of effect, *df*=5, *P*=.01.

^g^Italicized odds ratios (OR) denotes statistical significance.

^h^Overall test of effect, *df*=3, *P*=.40.

^i^Overall test of effect, *df*=3, *P*=.13.

^j^Overall test of effect, *df*=3, *P*=.16.

^k^PHQ-9: Patient Health Questionnaire 9-item scale.

^l^A score of ≥10 on the PHQ-9 indicates at least moderate depression severity.

^m^Higher scores indicate more positive attitudes toward seeking professional psychological help.

^n^Overall test of effect, *df*=3, *P*=.11.

^o^Overall test of effect, *df*=3, *P*=.10.

^p^Overall test of effect, *df*=3, *P*=.055.
