# Supplementary material for: Attitudes Toward Seeking Mental Health Services and Mobile Technology to Support the Management of Depression Among Black American Women: Cross-Sectional Survey Study
Source: J Med Internet Res. 2023 Jul 19;25:e45766. doi: 10.2196/45766 (PMC10398364; doi:10.2196/45766)
Supplement: Multimedia Appendix 6 [file jmir_v25i1e45766_app6.docx]

| **Multimedia Appendix 6. Significant interactions for a depression severity (Patient Health Questionnaire 9-item scale [PHQ-9]) cutoff score of 7 (0-6 vs 7-27) in multivariable logistic regression models for attitudes toward using each modality to communicate with a professional to receive support for managing depression.** | | | | |
| --- | --- | --- | --- | --- |
|  | Agree^a^ | Multi-variably adjusted^c^ OR (95% CI) at Depression Severity (PHQ-9)b Score 0-6 | Multi-variably adjusted^c^ OR (95% CI) at Depression Severity (PHQ-9)b Score 7-27 | p-value for interaction |
|  |  |  |  |  |
| **SMS Text Messaging** | | | | |
| **Psychological Openness^c^** | | | | |
| Score 0-16 | 27/48 (56.3%) | 0.68 (0.28-1.66) | 1.85 (0.70-4.87) | .14 |
| Score 17-32 | 226/346 (65.3%) | Ref | Ref |  |
|  |  |  |  |  |
| **Help-seeking Propensity^c^** | | |  |  |
| Score 0-16 | 14/28 (50%) | 0.74 (0.22-2.48) | 0.50 (0.15-1.64) | .65 |
| Score 17-32 | 239/366 (65.3%) | Ref | Ref |  |
|  |  |  |  |  |
| **Indifference to Depression Stigma^c^** |  |  |  |  |
| Score 0-16 | 25/48 (52.1%) | 0.72 (0.30-1.75) | **3.95 (1.34-11.67)** | *.02 ^d^* |
| Score 17-32 | 226/343 (65.9%) | Ref | Ref |  |
|  |  |  |  |  |
| **Voice Call** | | | | |
| **Psychological Openness^c^** | | | | |
| Score 0-16 | 27/48 (56.3%) | 0.98 (0.39-2.50) | 1.57 (0.53-4.66) | .52 |
| Score 17-32 | 226/346 (65.3%) | Ref | Ref |  |
|  |  |  |  |  |
| **Help-seeking Propensity^c^** | | |  |  |
| Score 0-16 | 14/28 (50%) | 0.35 (0.11-1.17) | 0.22 (0.07-0.72) | .57 |
| Score 17-32 | 239/366 (65.3%) | Ref | Ref |  |
|  |  |  |  |  |
| **Indifference to Depression Stigma^c^** |  |  |  |  |
| Score 0-16 | 25/48 (52.1%) | 0.70 (0.28-1.76) | 0.89 (0.33-2.44) | .73 |
| Score 17-32 | 226/343 (65.9%) | Ref | Ref |  |
| ` |  |  |  |  |
| **Mobile App** | | | | |
| **Psychological Openness^c^** | | | | |
| Score 0-16 | 27/48 (56.3%) | 0.87 (0.36-2.11) | 1.99 (0.73-5.40) | .23 |
| Score 17-32 | 226/346 (65.3%) | Ref | Ref |  |
|  |  |  |  |  |
| **Help-seeking Propensity^c^** | | |  |  |
| Score 0-16 | 14/28 (50%) | 0.75 (0.22-2.54) | 1.18 (0.36-3.91) | .60 |
| Score 17-32 | 239/366 (65.3%) | Ref | Ref |  |
|  |  |  |  |  |
| **Indifference to Depression Stigma^c^** |  |  |  |  |
| Score 0-16 | 25/48 (52.1%) | **0.23 (0.08-0.67)** | **3.19 (1.07-9.50)** | *<.001* |
| Score 17-32 | 226/343 (65.9%) | Ref | Ref |  |
|  |  |  |  |  |
| **Video Call** | | | | |
| **Psychological Openness^c^** | | | | |
| Score 0-16 | 27/48 (56.3%) | 0.59 (0.25-1.42) | 0.59 (0.23-1.55) | .996 |
| Score 17-32 | 226/346 (65.3%) | Ref | Ref |  |
|  |  |  |  |  |
| **Help-seeking Propensity^c^** | | |  |  |
| Score 0-16 | 14/28 (50%) | 0.45 (0.14-1.49) | 0.32 (0.10-1.04) | .69 |
| Score 17-32 | 239/366 (65.3%) | Ref | Ref |  |
|  |  |  |  |  |
| **Indifference to Depression Stigma^c^** |  |  |  |  |
| Score 0-16 | 25/48 (52.1%) | 0.98 (0.97-0.99) | 0.85 (0.49-1.48) | 0.59 |
| Score 17-32 | 226/343 (65.9%) | Ref | Ref |  |
|  |  |  |  |  |
| ^a^Agree indicates agreement with the use of text messaging to communicate with a professional to receive support for managing depression.  ^b^Score of 10 or higher on the Patient Health Questionnaire (PHQ-9) indicates at least moderate depression severity.  ^c^Higher scores indicate more positive attitudes toward seeking professional psychological help.  ^d^Italicized *P* value denotes statistical significance. | | | | |
