# Supplementary material for: Attitudes Toward Seeking Mental Health Services and Mobile Technology to Support the Management of Depression Among Black American Women: Cross-Sectional Survey Study
Source: J Med Internet Res. 2023 Jul 19;25:e45766. doi: 10.2196/45766 (PMC10398364; doi:10.2196/45766)
Supplement: Multimedia Appendix 7 [file jmir_v25i1e45766_app7.docx]

| **Multimedia Appendix 7. Significant interactions for a depression severity (Patient Health Questionnaire 9-item scale [PHQ-9]) cutoff score of 10 (0-9 vs 10-27) in multivariable logistic regression models for attitudes toward using each modality to communicate with a professional to receive support for managing depression.** | | | | |
| --- | --- | --- | --- | --- |
|  | Agree^a^ | Multi-variably adjusted^c^ OR (95% CI) at Depression Severity (PHQ-9)b Score 0-9 | Multi-variably adjusted^c^ OR (95% CI) at Depression Severity (PHQ-9)b Score 10-27 | p-value for interaction |
|  |  |  |  |  |
| **SMS Text Messaging** | | | | |
| **Psychological Openness^c^** | | | | |
| Score 0-16 | 27/48 (56.3%) | 0.88 (0.41-1.85) | 1.86 (0.50-6.83) | .33 |
| Score 17-32 | 226/346 (65.3%) | Ref | Ref |  |
|  |  |  |  |  |
| **Help-seeking Propensity^c^** | | |  |  |
| Score 0-16 | 14/28 (50%) | 0.72 (0.24-2.14) | 0.38 (0.09-1.49) | .47 |
| Score 17-32 | 239/366 (65.3%) | Ref | Ref |  |
|  |  |  |  |  |
| **Indifference to Depression Stigma^c^** |  |  |  |  |
| Score 0-16 | 25/48 (52.1%) | 1.41 (0.65-3.06) | 1.57 (0.47-5.29) | .89 |
| Score 17-32 | 226/343 (65.9%) | Ref | Ref |  |
|  |  |  |  |  |
| **Voice Call** | | | | |
| **Psychological Openness^c^** | | | | |
| Score 0-16 | 27/48 (56.3%) | 0.10 (0.98-1.01) | 0.82 (0.47-1.43) | .16 |
| Score 17-32 | 226/346 (65.3%) | Ref | Ref |  |
|  |  |  |  |  |
| **Help-seeking Propensity^c^** | | |  |  |
| Score 0-16 | 14/28 (50%) | 0.99 (0.98-1.01) | 0.91 (0.52-1.57) | .53 |
| Score 17-32 | 239/366 (65.3%) | Ref | Ref |  |
|  |  |  |  |  |
| **Indifference to Depression Stigma^c^** |  |  |  |  |
| Score 0-16 | 25/48 (52.1%) | 0.10 (0.98-1.01) | 0.83 (0.48-1.44) | .29 |
| Score 17-32 | 226/343 (65.9%) | Ref | Ref |  |
| ` |  |  |  |  |
| **Mobile App** | | | | |
| **Psychological Openness^c^** | | | | |
| Score 0-16 | 27/48 (56.3%) | 1.05 (0.50-2.23) | 2.24 (0.54-9.27) | .36 |
| Score 17-32 | 226/346 (65.3%) | Ref | Ref |  |
|  |  |  |  |  |
| **Help-seeking Propensity^c^** | | |  |  |
| Score 0-16 | 14/28 (50%) | 0.97 (0.33-2.85) | 0.69 (0.17-2.77) | .7 |
| Score 17-32 | 239/366 (65.3%) | Ref | Ref |  |
|  |  |  |  |  |
| **Indifference to Depression Stigma^c^** |  |  |  |  |
| Score 0-16 | 25/48 (52.1%) | 0.65 (0.29-1.44) | 1.13 (0.33-3.92) | .45 |
| Score 17-32 | 226/343 (65.9%) | Ref | Ref |  |
|  |  |  |  |  |
| **Video Call** | | | | |
| **Psychological Openness^c^** | | | | |
| Score 0-16 | 27/48 (56.3%) | 0.90 (0.42-1.93) | **0.19 (0.05-0.68)** | *.04 ^d^* |
| Score 17-32 | 226/346 (65.3%) | Ref | Ref |  |
|  |  |  |  |  |
| **Help-seeking Propensity^c^** | | |  |  |
| Score 0-16 | 14/28 (50%) | 0.39 (0.13-1.15) | 0.35 (0.09-1.36) | .88 |
| Score 17-32 | 239/366 (65.3%) | Ref | Ref |  |
|  |  |  |  |  |
| **Indifference to Depression Stigma^c^** |  |  |  |  |
| Score 0-16 | 25/48 (52.1%) | 0.52 (0.24-1.14) | 0.26 (0.08-0.874) | .34 |
| Score 17-32 | 226/343 (65.9%) | Ref | Ref |  |
|  |  |  |  |  |
| ^a^Agree indicates agreement with the use of text messaging to communicate with a professional to receive support for managing depression.  ^b^Score of 10 or higher on the Patient Health Questionnaire (PHQ-9) indicates at least moderate depression severity.  ^c^Higher scores indicate more positive attitudes toward seeking professional psychological help.  ^d^Italicized *P* value denotes statistical significance. | | | | |
